# Transglutaminase 2 regulates vimentin-dependent proteostasis during macrophage activation

**DOI:** 10.1126/sciadv.aea7513

**Published:** 2026-08-28

**Authors:** Yali Xu, Ting Su, Hricha Mishra, Naoshi Dohmae, Takehiro Suzuki, Yuriko Sakamaki, Haruyo Aoyagi, Hideki Aizaki, Hajime Nishimura, Erina Furuhata, Yuqing Gong, Qi Cheng, Beiyuan Zhang, Chenzhe He, Kaori Yanaka, Yutaka Furutani, Hideki Tatsukawa, Harukazu Suzuki, Wenkui Yu, Xian-Yang Qin

**Affiliations:** ^1^ Department of Intensive Care Unit, Affiliated Drum Tower Hospital, Medical School of Nanjing University, Gulou, Nanjing 210008, China.; ^2^ Laboratory for Cellular Function Conversion Technology, RIKEN Center for Integrative Medical Sciences, Tsurumi-ku, Yokohama 2300045, Japan.; ^3^ Biomolecular Characterization Unit, Technology Platform Division, RIKEN Center for Sustainable Resource Science, Hirosawa, Wako 3510106, Japan.; ^4^ Ochanomizu Research Facility (ORF), Bioscience Center, Institute of Science Tokyo, Bunkyo-ku, Tokyo 1138510, Japan.; ^5^ Department of Virology II, Japan Institute for Health Security, Tokyo 1628640, Japan.; ^6^ Department of Laboratory Medicine, The Jikei University School of Medicine, Minato-ku, Tokyo 1058461, Japan.; ^7^ Department of Basic Medicinal Sciences, Graduate School of Pharmaceutical Sciences, Nagoya University, Chikusa-ku, Nagoya 4648601, Japan.; ^8^ Department of Gastroenterology, Graduate School of Medicine, Gifu University, Yanagido, Gifu 5011194, Japan.

## Abstract

Sepsis-associated liver dysfunction is a life-threatening condition with a high mortality rate and no mechanism-based therapy. In this study, we identify the cross-linking enzyme transglutaminase 2 (TG2) as a driver of liver inflammation by activating macrophages in a mouse model of sepsis. Pharmacological inhibition of TG2 improves survival and reduces multiorgan inflammation, with the liver as a primary therapeutic target. Mechanistically, TG2 activity was up-regulated in macrophages, where it cross-linked vimentin to promote oligomerization and intermediate filament remodeling. Genetic ablation of TG2 or vimentin suppressed macrophage cytokine production and attenuated lipopolysaccharide-induced inflammation. Notably, vimentin-deficient macrophages exhibited enhanced proteasome recruitment to detergent-insoluble protein aggregates, accelerating the degradation of proinflammatory mediators such as Traf6, thereby dampening nuclear factor κB signaling. Proteomic profiling revealed a previously unrecognized Rab27a-positive vesicle trafficking pathway for inflammatory aggregate clearance. Together, these findings define a TG2–vimentin axis that controls macrophage activation through proteostasis regulation, linking cytoskeletal remodeling to inflammatory signaling.

## INTRODUCTION

In 2017, the World Health Organization recognized sepsis as a global health priority, requiring urgent advances in mechanistic understanding and therapeutic strategies. The first global sepsis report in 2020 estimated that sepsis was responsible for approximately 11 million deaths annually, accounting for 20% of all mortalities and surpassing cancer as the leading cause of mortality. The burden is particularly severe in older adults, where sepsis frequently progresses to irreversible septic shock, with mortality rates of up to 40% (*1*, *2*). Sepsis lethality is primarily driven by a dysregulated host immune response that results in widespread inflammation and multiorgan failure (*3*, *4*). The liver plays a central role in metabolic regulation and immune surveillance, making it particularly vulnerable to septic injuries. Sepsis-associated liver dysfunction (SALD) exacerbates the systemic inflammatory response and is an independent predictor of poor clinical outcomes (*5*, *6*). Despite significant advances in critical care, SALD remains a therapeutic blind spot with a mortality rate of 61.5%, far exceeding that of other sepsis-related organ failures. Current SALD management strategies adapted from other hepatopathies show limited efficacy owing to an insufficient understanding of disease-specific mechanisms. Our recent autopsy analyses demonstrated that, unlike ischemic or toxic liver injury, SALD exhibits distinct pathological features such as microarchitectural collapse of the bile canaliculi and a strong association with intra-abdominal infection and persistent gut microbiota dysbiosis (*7*, *8*). These findings underscore SALD as a unique pathophysiological state at the gut-liver interface. A deeper understanding of its mechanisms is urgently needed to overcome the critical barriers to targeted therapy development.

Macrophages are essential for orchestrating the initiation and amplification of organ-specific injury signals (*9*). Upon recognition of pathogen-associated molecular patterns such as lipopolysaccharide (LPS), double-stranded RNA, and flagellin, macrophages are activated via Toll-like receptor (TLR)–mediated signaling pathway, leading to robust production of proinflammatory cytokines, including tumor necrosis factor–α (TNF-α), interleukin-6 (IL-6), and IL-1β (*9*). The magnitude of this cytokine surge is directly associated with the severity of hepatic injury and early sepsis–related mortality (*10*). While preclinical studies have shown that neutralizing TNF, IL-6, and IL-1β can confer protection against tissue damage and improve survival, clinical trials targeting individual cytokines or upstream recognition pathways have failed to yield effective therapies (*11*, *12*). An integrated view of the coordinated pathophysiological mechanisms driving proinflammatory cytokine production may facilitate the optimization of organ injury management and help identify critical targets for innovative therapeutic strategies.

Transglutaminase 2 (TG2) is the most ubiquitously expressed member of the transglutaminase family, a class of enzymes that catalyze posttranslational modifications by targeting the γ-carboxamide group of glutamine residues (*13*). Its transamidase activity enables the formation of covalent ε(γ-glutamyl) lysine isopeptide bonds between glutamine and lysine residues, thereby mediating protein cross-linking (*14*). TG2-mediated cross-linking profoundly affects substrate proteins by altering their stability, solubility, and structural conformation. These modifications play critical roles in various physiological processes and pathological contexts (*15*). In neurodegenerative diseases, elevated TG2 activity promotes the aggregation of misfolded proteins such as amyloid-β, tau, and huntingtin, thereby contributing to neuronal toxicity. Similarly, TG2-driven cross-linking of cytokines (e.g., IL-2) and viral proteins has been shown to modulate immune responses and affect cellular viability or viral replication (*16*, *17*). Moreover, TG2 facilitates the covalent incorporation of trappin family proteins into the extracellular matrix, thereby enhancing mucosal antimicrobial defense. Our group has made foundational contributions to the understanding of the critical role of TG2 in liver injury and inflammation (*13*, *18*). We demonstrated that in multiple liver disease models, nuclear TG2 induces hepatocyte apoptosis by cross-linking and inactivating transcription factors (TFs), such as Sp1, leading to the suppression of downstream cell growth signaling pathways (*19*–*21*). Building on this, we developed an in situ method for detecting TG2 enzymatic activity in tissue sections using the TG2 substrate probe 5-biotinamidopentylamine (5BAPA). In a pilot study, we showed that TG2 activity dose-dependently increased in the midzonal liver macrophages of septic mice subjected to cecal ligation and puncture or LPS challenge (*22*). Notably, no BAPA signal was detected in the normal liver tissue despite detectable TG2 protein expression, indicating that TG2 enzymatic activity, rather than expression, is tightly regulated and specifically induced under inflammatory conditions. These findings suggest that TG2 activation is a distinct molecular event that occurs during sepsis progression and may represent both a mechanistic driver and a promising therapeutic target in SALD.

Proteins serve as primary functional molecules in cellular systems, and their activity is regulated through multiple layers of control, including expression levels, three-dimensional conformation, posttranslational modifications, and protein-protein interactions. Recent findings suggest that proteostasis, which governs protein synthesis, folding, transport, and degradation, is a key regulator of the inflammatory response. In macrophages, LPS stimulation enhances protein biosynthesis and drives the production of proinflammatory cytokines and major histocompatibility complex class II molecules (*23*). This abrupt elevation in protein synthesis imposes significant stress on the cellular proteostasis network, potentially leading to the accumulation of misfolded proteins and the subsequent formation of protein aggregates (AGGs). Such AGGs may exhibit cytotoxic effects by interfering with normal cellular processes or exert cytoprotective functions by sequestering damaged proteins into defined subcellular compartments (*24*). In mammalian cells, clearance of misfolded proteins is primarily mediated by two complementary systems: the ubiquitin-proteasome system (UPS), which degrades misfolded proteins, and the autophagy-lysosome pathway, which eliminates larger protein AGGs and damaged organelles (*25*, *26*). In addition, the unfolded protein response serves as a compensatory mechanism by enhancing molecular chaperone expression and protein folding capacity. Different cell types use specialized adaptations of these quality control mechanisms. For instance, hematopoietic stem cells preferentially compartmentalize misfolded proteins into AGGs that are subsequently cleared via aggrephagy (*27*). However, specific strategies used by macrophages to manage proteostatic stress during sepsis remain poorly understood.

Here, we systematically investigated the role of TG2 in this process using genetic and pharmacological approaches in a murine model of sepsis. We identified TG2-dependent vimentin (Vim) cross-linking as a key molecular event that disrupts macrophage proteostasis, leading to pathological protein aggregation and sustained inflammatory signaling. Our findings establish the TG2-Vim axis as a key mechanism linking cytoskeletal remodeling to inflammatory responses in sepsis.

## RESULTS

### Organ-dependent protective effect of TG2 inhibition in septic mice

**Fig. 1.**
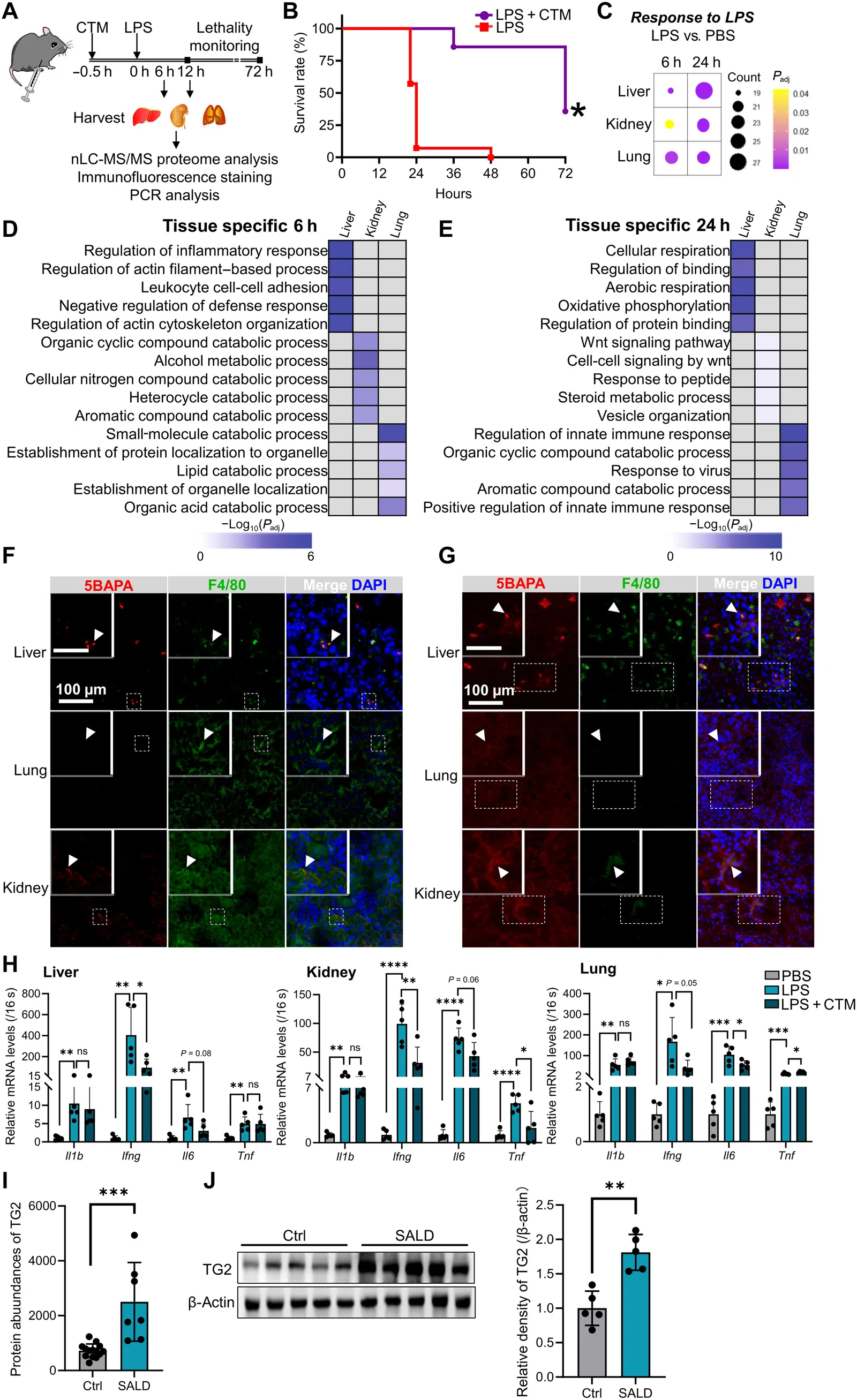
Organ-dependent protective effect of TG2 inhibition in septic mice. (**A**) Experimental design for CTM treatment in LPS-induced septic mice. h, hours. (**B**) Survival curves of mice treated with LPS (30 mg/kg) ± CTM (100 mg/kg). *P < 0.05 (log-rank test; *n* = 14 per group). (**C** to **E**) GO enrichment of (C), the Response to LPS term, and (D and E) tissue-specific biological process enrichment based on proteomic profiling at 6 hours (D) and 24 hours (E) post-LPS administration. (**F** and **G**) Triple immunofluorescence staining for 4′,6-diamidino-2-phenylindole (DAPI, blue), F4/80 (green, macrophages), and 5BAPA (red, TG2 activity) in (F) young and (G) aged mice. Arrowheads indicate 5BAPA^+^ macrophages. Insets, 20-μm scale. (**H**) Relative gene expression of inflammatory genes in young mice at 6 hours after LPS treatment. (**I**) TG2 protein abundance in human liver tissues identified by quantitative proteomics. (**J**) (Left) Immunoblots and (Right) bar graph of TG2 in human liver tissues. Data are presented as means ± SD; *P < 0.05, **P < 0.01, ***P < 0.001, ****P < 0.0001, Student’s *t* test; ns, not significant.

To evaluate whether pharmacological inhibition of TG2 can provide a systemic therapeutic benefit in sepsis, we treated LPS-induced septic mice with a TG2 inhibitor, cystamine (CTM), and assessed their survival and multiorgan responses (Fig. 1A). CTM treatment significantly improved survival outcomes in mice with sepsis (Fig. 1B), confirming its protective efficacy in vivo. Consistent with this, CTM treatment significantly reduced serum levels of the proinflammatory cytokines TNF-α (fig. S1A) and IL-6 (fig. S1B), as well as lactate dehydrogenase, a marker of tissue injury (fig. S1C). To delineate the organ-specific effects of CTM, we performed proteomic profiling of the liver, kidney, and lung tissues at early (6 hours) and late (24 hours) time points after LPS injection (data S1). Gene Ontology (GO) analysis of proteins up-regulated by LPS revealed strong enrichment of the “response to LPS” term across all three organs (Fig. 1C), highlighting the systemic nature of the inflammatory response (*28*, *29*). To identify CTM-regulated pathways, we analyzed the proteins modulated by CTM using the following criteria: [LPS/phosphate-buffered saline (PBS) > 2 and CTM/PBS < 0.8 × LPS/PBS) or (LPS/PBS < 0.5 and CTM/PBS > 1.2 × LPS/PBS]. At 6 hours post-LPS, CTM primarily altered lipid metabolism–related processes across all three organs, whereas at 24 hours, pathways related to endopeptidase activity and cytochrome c release were more prominently affected (Fig. 1D). CTM selectively influenced the inflammatory and cytoskeletal organization pathways in the liver at 6 hours (Fig. 1E) and modulated the protein-binding and mitochondrial respiration pathways at 24 hours (fig. S1D), with no comparable effects were observed in the lungs or kidneys. Ingenuity pathway analysis (IPA) further revealed that CTM down-regulated canonical inflammation–related pathways, specifically in the liver, at both time points (fig. S1E). To assess tissue-specific TG2 activation, mice were intraperitoneally injected with the biotinylated TG2 substrate 5BAPA 30 min before euthanasia, followed by tetramethylrhodamine isothiocyanate (TRITC)–streptavidin staining. Consistent with our previous findings (*22*), LPS induced robust TG2 activation in F4/80-positive hepatic macrophages, whereas little or no signal was detected in the lung or kidney macrophages, indicating liver-specific TG2 activation during sepsis (Fig. 1F). The same liver-restricted pattern was evident in the 16-month-old mice (Fig. 1G). Polymerase chain reaction (PCR) analysis further demonstrated that CTM reduced the expression of proinflammatory cytokines (e.g., *Ifng* and *Il6*) in the liver, kidneys, and lungs of both young and aged mice, with the most pronounced suppression observed in the livers of aged animals (Fig. 1H and fig. S1F). These results demonstrate the beneficial effect of the pharmacological TG2 inhibition by CTM in sepsis, with the liver acting as the primary therapeutic target. Last, we examined the clinical relevance of TG2 in sepsis. Data mining of publicly available RNA sequencing (RNA-seq) datasets of multiorgan tissues from patients with meningococcal septic shock (*30*) revealed a significant up-regulation of *Tgm2* mRNA in the liver and heart, but not in the kidney, lung, or spleen, compared with noninfectious controls (fig. S1G). In addition, quantitative proteomics (Fig. 1I) and Western blot analysis (Fig. 1J) demonstrated a marked increase in TG2 protein abundance in the livers of patients with SALD compared with noninfectious controls. Together, these data suggested a potential contribution of TG2 in the liver to sepsis pathophysiology.

### Functional validation of macrophage-dependent TG2 activity in septic mice

**Fig. 2.**
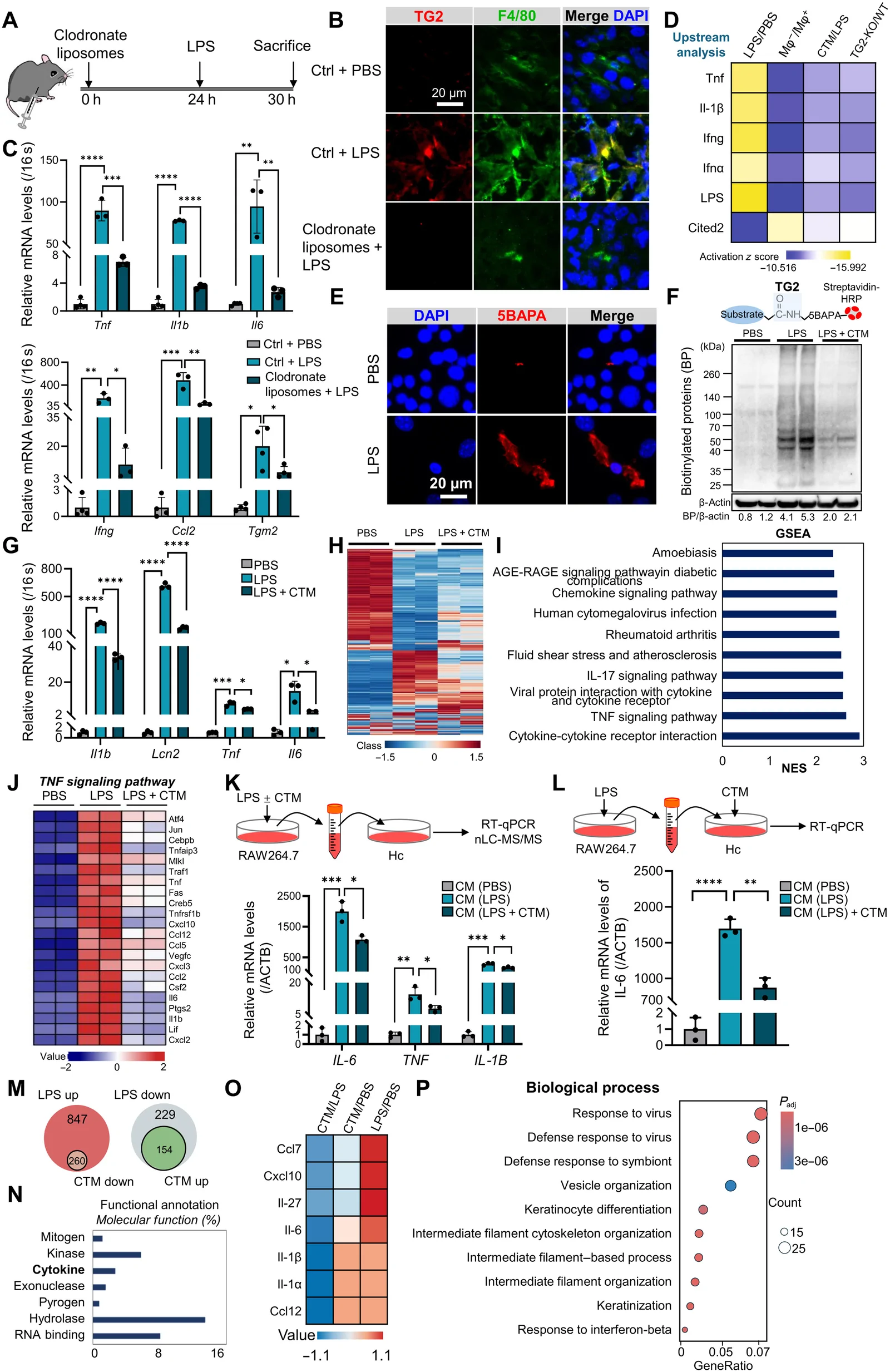
TG2 transamidase activity drives macrophage inflammatory responses. (**A**) Experimental scheme for macrophage depletion in LPS-induced sepsis. (**B**) Triple immunofluorescence staining of DAPI (blue), F4/80 (green), and TG2 (red) in liver sections. (**C**) Hepatic expression of inflammatory genes and *Tgm2*. (**D**) Top predicted upstream regulators of DEGs identified by RNA-seq, ranked by activation *z* scores (IPA). Mφ, macrophage. (**E**) Immunofluorescence staining of DAPI (blue) and 5BAPA (red) in LPS-treated RAW264.7 cells. (**F**) Immunoblots of biotinylated proteins labeled by TG2-catalyzed 5BAPA incorporation. (**G**) Expression of inflammatory genes in LPS-treated RAW264.7 cells with or without CTM. (**H**) Hierarchical clustering of RNA-seq profiles. (**I**) GSEA of LPS-regulated genes ± CTM. NES, normalized enrichment score. (**J**) Heatmap of genes in the “TNF signaling pathway.” (**K**) Effect of CM from LPS-treated RAW264.7 cells ± CTM on hepatocytes (Hc cells). (**L**) Direct effect of CTM on Hc cells exposed to LPS-stimulated CM. (**M**) Nested circle plots of CTM-regulated proteins in CM identified by nLC-MS/MS. (**N**) UniProtKB keyword enrichment of CTM-regulated proteins. (**O**) Heatmap of CTM-regulated cytokines. (**P**) GO biological process enrichment of CTM-regulated proteins. Data are presented as means ± SD; *P < 0.05, **P < 0.01, ***P < 0.001, ****P < 0.0001; Student’s *t* test.

To verify the functional role of macrophages in LPS-induced sepsis, macrophage depletion was achieved using clodronate liposomes (*31*), followed by an LPS challenge (Fig. 2A). Intraperitoneal administration of clodronate liposomes effectively reduced macrophage abundance in the liver but not in the lungs or kidneys, as evidenced by decreased *Adgre1* expression (fig. S2A), and reduced F4/80 immunofluorescence staining (Fig. 2B). Hepatic macrophage depletion markedly suppressed LPS-induced expression of inflammatory cytokines, including *Tnf*, *Il1b*, *Il6*, *Ifng*, and *Ccl2* (Fig. 2C). Given our observation that macrophages serve as the source of activated TG2 following LPS treatment, the depletion of hepatic macrophages abolished TG2 signaling in F4/80-positive cells (Fig. 2B) and significantly reduced *Tgm2* expression in the liver (Fig. 2C). To further elucidate the functional relationship between TG2 and macrophages, we performed RNA-seq of liver tissues from LPS-challenged wild-type (WT) mice treated with CTM, TG2 knockout (TG2-KO) mice, and macrophage-depleted mice (data S2). Differentially expressed genes (DEGs) (fold change > 2) were subjected to IPA. Upstream regulator analysis revealed that key inflammatory mediators, including Il-1β, interferon, and Tnf, exhibited similarly suppressed activation z-scores across all three conditions (Fig. 2D). These data support the notion that TG2 and hepatic macrophages function as coordinated effectors of LPS signaling and are critical drivers of inflammatory responses in the liver during sepsis.

### TG2 transamidase activity drives macrophage inflammatory responses

To determine whether TG2 activation observed in vivo reflects an intrinsic macrophage response rather than the uptake of 5BAPA-positive debris, we stimulated RAW264.7 cells with LPS in vitro. LPS-induced TG2 transamidase activity in RAW264.7 cells, evidenced by enhanced 5BAPA incorporation detected by TRITC-streptavidin microscopy (Fig. 2E) and immunoblotting (Fig. 2F). This activation was almost completely abolished by CTM (Fig. 2F and fig. S2B), indicating the direct LPS-dependent activation of TG2 in macrophages and its efficient suppression by CTM. PCR analysis and fluorescence-based detection using the reactive oxygen species (ROS) probe 5–6-chloromethyl-2′,7′-dichlorodihydrofluorescein diacetate (CM-H_2_DCFDA) revealed increased TG2 expression (fig. S2C) and elevated intracellular ROS levels (fig. S2D) in LPS-stimulated macrophages. Consistent with our previous finding that pathogenic fungi activate TG2 through ROS production (*21*), these results suggest that LPS might enhance both TG2 abundance and an ROS-rich intracellular environment permissive for TG2 activation. Functionally, CTM attenuated LPS-induced expression of inflammatory cytokines, including *Il1b*, *Lcn2*, *Tnf*, and *Il6* in RAW264.7 cells (Fig. 2G). Heatmap analysis (Fig. 2H) of the RNA-seq data confirmed that CTM broadly suppressed LPS-induced inflammatory transcriptional programs in macrophages (data S3). Among the 1250 DEGs (fold change > 2) upon LPS stimulation, CTM down-regulated and up-regulated 711 and 539 transcripts, respectively. Gene set enrichment analysis (GSEA), performed on genes ranked by ascending fold change regulated by CTM, revealed significant attenuation of proinflammatory signaling pathways (Fig. 2I), including TNF signaling (Fig. 2J), IL-17 signaling (fig. S2E), and cytokine–cytokine receptor interactions (fig. S2F). To rule out LPS-independent effect of CTM, we performed additional RNA-seq analyses to compare transcriptional profiles under PBS, CTM-alone, LPS-alone, and combined LPS + CTM treatment conditions (fig. S2G). Heatmap analysis showed that genes differentially expressed between LPS and LPS + CTM conditions exhibited highly similar baseline expression patterns in the PBS and CTM-alone groups, suggesting that CTM does not substantially alter the resting-state expression of LPS-responsive genes (fig. S2H). To further dissect CTM-specific effects and its interaction with LPS, we applied a three-component linear deconvolution model, as previously described (*32*). This framework conceptualizes gene expression changes as the sum of three regulatory components: (i) LPS-dependent regulation independent of CTM (LPS), (ii) CTM-dependent regulation independent of LPS (CTM), and (iii) synergistic or antagonistic interactions between LPS and CTM (INT). Among 2056 DEGs, approximately 87% were well explained by this model (*R*^2^ > 0.9). Of these, 1706 genes contained at least one significant regulatory component (*P* < 0.05) and were classified into 17 clusters (>5 genes). Clusters 1, 3, 5, 6, 14, and 17 represented genes antagonistically regulated by LPS and CTM. These clusters included key inflammatory cytokines (*Il6*, *Il1a*, *Il1b*, and *Il27*), chemokines (*Ccl2*, *Ccl3*, *Ccl4*, *Ccl5*, *Cxcl2*, *Cxcl3*, and *Cxcl5*), and acute-phase response genes (*Oas1/2/3* and *Saa3*) (fig. S2I). Although both LPS and CTM independently induced the expression of these genes, their combined treatment resulted in a pronounced antagonistic effect, leading to attenuated inflammatory gene expression. In contrast, clusters 8, 10 to 13, 15, and 16 comprised genes independently regulated by LPS or CTM. Last, clusters 2, 4, 7, and 9 contained genes synergistically regulated by LPS and CTM, including multiple regulators of cell-cycle progression (*Noxo1*, *Cdk1*, *Ccna2*, *Ccnb1/2*, *Kif22*, *Kif2c*, *Kif20a*, and *Kif23*) (fig. S2I). Functional enrichment analysis further supported these distinctions. CTM-induced, LPS-insensitive genes (CTM^up^/LPS^IN^) were uniquely enriched in pathways related to responses to toxic substances, oxidative stress, and phagocytosis, highlighting CTM’s standalone cellular effects (fig. S2J). In contrast, LPS-synergistic genes (LPS^SYN^) were enriched in pathways associated with positive regulation of innate immune responses, responses to bacterial molecules, and TNF family cytokine production (fig. S2J). Notably, although inflammatory cytokines and nuclear factor κB (NF-κB) regulators could be independently activated by either LPS or CTM, their combined treatment resulted in a dominant antagonistic interaction that suppressed inflammatory gene expression (fig. S2K). Together, these results demonstrate that CTM activates a transcriptional program that is molecularly distinct from that induced by LPS and antagonizes LPS-driven inflammatory gene expression through a defined interaction component rather than through off-target activation of inflammatory pathways. Moreover, we used NC9, an irreversible and selective TG2 inhibitor, and generated TG2-deficient RAW264.7 cells via CRISPR-Cas9. Both NC9 treatment and TG2-KO recapitulated the anti-inflammatory effects of CTM, as evidenced by the reduced expression of inflammatory cytokines such as *Il1b* and *Il6* (fig. S2, L to N). Furthermore, cell sorting showed that 5BAPA high macrophages expressed more *Il1b* than did 5BAPA low cells (fig. S2O), directly linking TG2 activity to cytokine expression. To evaluate the role of TG2 within liver macrophages in hepatic inflammation in vivo, we used an adapted macrophage depletion and reconstitution model that combines depletion of resident macrophages using clodronate liposomes followed by intravenous reconstitution with RAW264.7 cells (fig. S2P) (*33*). Upon LPS challenge, mice receiving TG2-deficient macrophages exhibited significantly reduced hepatic expression of *Il6*, along with modest reductions in *Tnf* and *Il1b*, compared with mice reconstituted with WT macrophages (fig. S2Q).

### TG2 transamidase activity drives macrophage-hepatocyte cross-talk

Given that CTM treatment alleviated LPS-induced liver injury in vivo, we investigated macrophage-hepatocyte cross-talk using a conditioned medium (CM) derived from LPS-stimulated RAW264.7 macrophages. When applied to human hepatocyte Hc cells, CM from LPS + CTM–treated macrophages significantly reduced expression of inflammatory cytokines *IL-6*, *TNF*, and *IL-1B* compared to CM from LPS alone–treated macrophages (Fig. 2K). Notably, the direct addition of CTM to the CM from LPS-activated macrophages also suppressed *IL-6* expression in hepatocytes (Fig. 2L), raising the possibility that intrinsic TG2 activity in hepatocytes may contribute to their inflammatory response. Quantitative proteomic profiling of the macrophage-derived CM identified 414 proteins modulated by CTM, 2.5% of which were cytokines (data S4 and Fig. 2, M and N). Notably, CTM markedly reduced the levels of secreted proinflammatory mediators, including Ccl7, Cxcl10, Il-6, and Ccl12 (Fig. 2O). GO enrichment analysis of the CTM-responsive secretome components revealed significant associations with biological processes, such as vesicle organization and intermediate filament organization; cellular components, including the aggresome and intermediate filament cytoskeleton; and molecular functions, such as ubiquitin-like protein ligase binding (Fig. 2P and fig. S2R). Collectively, these findings demonstrate that LPS directly activates TG2 in macrophages and that both pharmacological and genetic inhibition of TG2 attenuates macrophage inflammatory responses and suppresses macrophage-to-hepatocyte inflammatory signaling by modulating the macrophage secretome.

### Vim is a TG2 substrate in activated macrophages

**Fig. 3.**
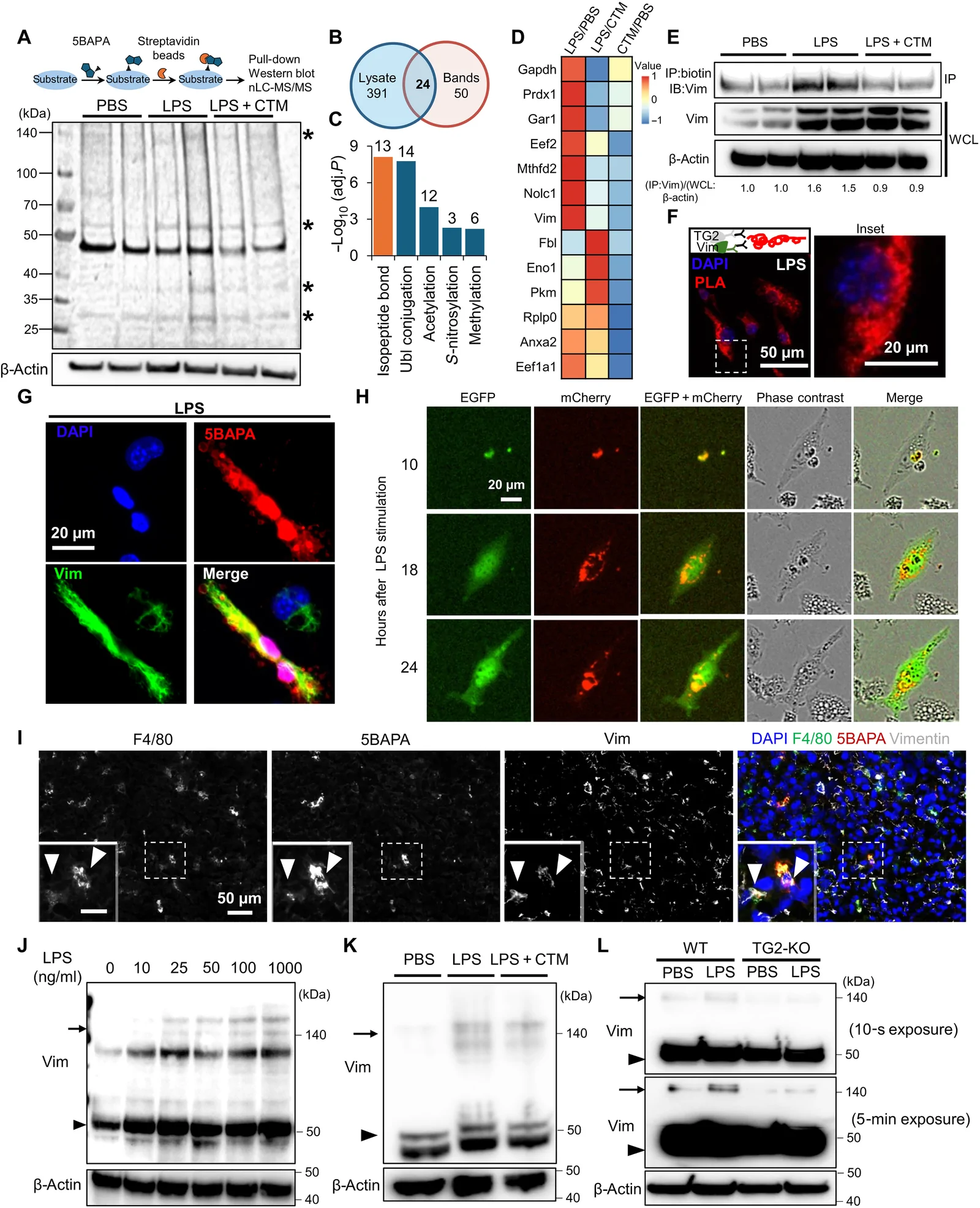
Vim is a TG2 substrate in activated macrophages. (**A**) (Top) Experimental design for TG2 substrate identification based on 5BAPA biotinylation in RAW264.7 cells. (Bottom) Silver-stained SDS-PAGE of streptavidin pull-down fractions. Asterisks indicate LPS-enhanced bands attenuated by CTM. (**B**) Venn diagram of proteins identified by nLC-MS/MS from excised gel bands and total eluates. (**C**) Posttranslational modification annotation of the 24 overlapping candidates based on UniProt. The –log_10_ (adjusted *P* value) is shown on the *y* axis. Gene counts are indicated at the top of each bar. (**D**) Heatmap of normalized abundance of proteins annotated with the Uniprot keyword Isopeptide bond. (**E**) Immunoblots of Vim in whole-cell lysates (WCL) and streptavidin pull-downs. (**F**) PLA (red) detecting Vim-TG2 interactions in LPS-treated RAW264.7 cells. (**G**) Triple immunofluorescence staining for DAPI (blue), Vim (green), and 5BAPA (red) in RAW264.7 cells stimulated with LPS (1 μg/ml) for 24 hours. (**H**) Time-lapse imaging of LPS-treated RAW264.7 cells coexpressing EGFP-TG2 (green) and mCherry-Vim (red, 20× objective). (**I**) Quadruple immunofluorescence staining for DAPI (blue), F4/80 (green, macrophages), 5BAPA (red), and Vim (gray) in livers of LPS-induced septic mice. (**J**) Immunoblots of Vim in RAW264.7 cells treated with increasing LPS doses. (**K**) Immunoblots of Vim in LPS-treated RAW264.7 cells ± CTM. (**L**) Immunoblots of Vim in LPS-treated WT and TG2-KO RAW264.7 cells. Arrowheads indicate monomeric Vim; arrows indicate oligomers.

To elucidate the mechanism by which CTM treatment mitigates the inflammatory response, we sought to identify TG2 substrates in LPS-stimulated RAW264.7 macrophages using a streptavidin-biotin affinity pull-down strategy coupled with nano liquid chromatography–tandem mass spectrometry (nLC-MS/MS). The TG2 probe 5BAPA, which is incorporated into the glutamine residues of TG2 substrates, enables the selective biotinylation of transamidated proteins. Streptavidin-conjugated beads were then used to enrich biotinylated proteins from lysates of RAW264.7 cells treated with PBS, LPS, or LPS + CTM (Fig. 3A, top). The eluted proteins were either analyzed directly by nLC-MS/MS (data S5), or resolved by SDS–polyacrylamide gel electrophoresis (SDS–PAGE). Following visualization by silver staining (Fig. 3A, bottom), four protein bands (marked with asterisks) that were up-regulated upon LPS stimulation and were reversed by CTM treatment were excised and subjected to nLC-MS/MS analysis (data S6). Overall, 24 candidate TG2 substrates were consistently identified across both proteomic approaches, showing increased abundance upon LPS stimulation and attenuation by CTM (Fig. 3B). Of these, 13 proteins were significantly annotated with the UniProt keyword “Isopeptide bond,” a posttranslational modification associated with TG2 activity (Fig. 3, C and D). Among these candidates, we focused on the intermediate filament protein Vim because of prior evidence of its TG2-mediated modification in the bovine lens (*34*) and its emerging role in macrophage-driven inflammation (*35*). To validate Vim as a TG2 substrate, we immunoprecipitated biotinylated proteins from LPS-treated RAW264.7 and performed immunoblotting with an anti-Vim antibody. A strong Vim signal was detected in the pull-down assay of LPS-treated cells, which was markedly reduced by CTM treatment (Fig. 3E and fig. S3A), confirming that Vim was a transamidation target of TG2 in activated macrophages. Notably, core histones (H2A, H2B, H3, and H4), which are known glutaminyl substrates of TG2 (*36*), were not identified among proteins enriched by LPS stimulation and reversed by CTM treatment in our biotinylated proteome analysis (fig. S3B).

### TG2 catalyzes the oligomerization of Vim in activated macrophages

To further investigate the interaction between TG2 and Vim, we used a proximity ligation assay (PLA), which generates a fluorescent signal when the two proteins are within 40 nm. PLA performed using anti-TG2 and anti-Vim antibodies revealed strong signals in LPS-treated RAW264.7 cells, indicating a close spatial association between TG2 and Vim under inflammatory conditions (Fig. 3F). Supporting this finding, immunofluorescence staining showed marked colocalization of Vim with 5BAPA-labeled TG2 substrates in LPS-stimulated macrophages (Fig. 3G). Live-cell imaging of RAW264.7 cells coexpressing enhanced green fluorescent protein (EGFP)–TG2 and mCherry-Vim further demonstrated dynamic aggregation and colocalization of the two proteins upon LPS treatment, suggesting their functional interaction during macrophage activation (Fig. 3H). In vivo, immunofluorescence analysis of LPS-treated mouse liver tissue revealed colocalization of Vim with 5BAPA incorporation within F4/80^+^ macrophages, consistent with TG2-mediated transamidation of Vim in hepatic macrophages (Fig. 3I). This signal was markedly diminished following macrophage depletion, indicating that liver macrophages were the predominant source of TG2-Vim interaction during inflammation (fig. S3C). Moreover, LPS-induced Vim expression was abolished by macrophage depletion (fig. S3D), further supporting a macrophage-specific response. Western blot analysis revealed that LPS stimulation induced the formation of higher-order Vim oligomers in a dose-dependent manner, appearing as bands at approximately 140 kDa (Fig. 3J). Notably, LPS-induced Vim oligomerization was substantially suppressed by CTM treatment (Fig. 3K) and was abrogated in TG2-KO RAW264.7 cells (Fig. 3L), highlighting the essential role of TG2 enzymatic activity in driving Vim polymerization during macrophage-mediated inflammation. SDS-PAGE analysis indicated that only a small fraction of total Vim is detectably cross-linked by TG2. It is possible that TG2 introduces covalent ε-(γ-glutamyl)-lysine bonds, which are stable under denaturing conditions and therefore detectable by SDS-PAGE, whereas native Vim intermediate filaments are assembled through noncovalent interactions that are disrupted during electrophoresis (*37*). As a proof of concept, we performed native PAGE to assess higher-order Vim organization and observed an increase in high–molecular weight Vim species, together with a shift from lower- to higher-order assemblies following LPS stimulation (fig. S3E). These observations suggest that covalent modification of a limited subset of dynamic Vim molecules by TG2 may be sufficient to influence higher-order assembly states within a predominantly noncovalent filament network, potentially leading to structural remodeling.

### Vim drives macrophage inflammatory responses

**Fig. 4.**
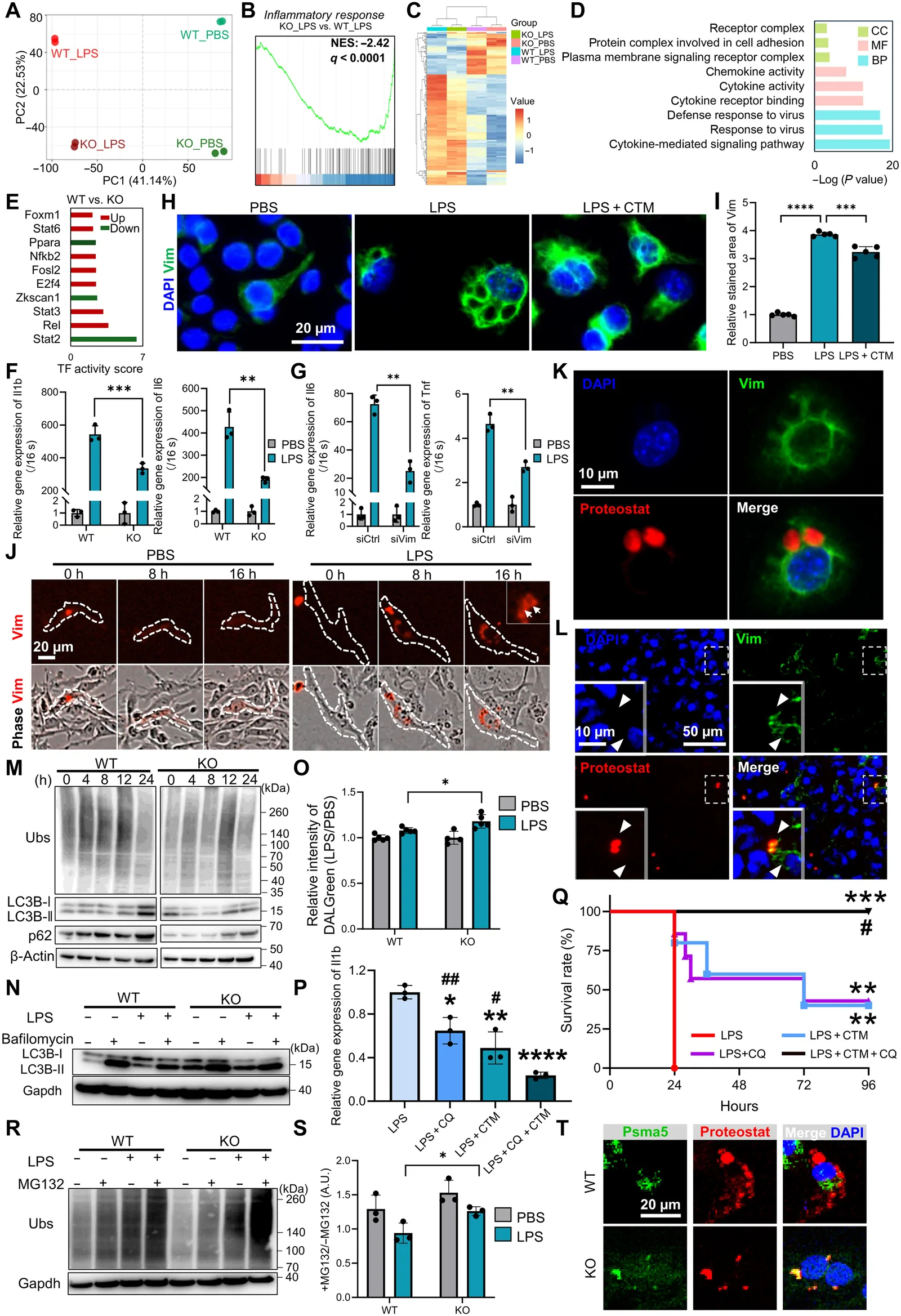
Vim mediates proteasome-dependent proteostasis. (**A**) PCA of RNA-seq profiles from WT and Vim-KO RAW264.7 macrophages treated with PBS or LPS (1 μg/ml, 12 hours). (**B**) GSEA for the inflammatory response term in the dataset from (A). NES, normalized enrichment score. (**C**) Heatmap of LPS-responsive DEGs. (**D**) GO enrichment analysis of DEGs across functional categories. (**E**) Predicted TF activities from VIPER analysis. (**F** and **G**) Relative expression of inflammatory cytokines *Il1b*, *Il6*, and *Tnf* in (F) WT/KO cells and (G) cells transfected with control or Vim-targeting siRNA. (**H** and **I**) Immunofluorescence (IF) of Vim (Vim, green) in cells treated with LPS ± CTM and its quantification. (**J**) Time-lapse imaging of mCherry-Vim during LPS treatment. (**K** and **L**) Triple-IF for DAPI (blue), Proteostat (AGGs, red), and Vim (green) in (K) LPS-treated cells and (L) livers from septic mice. (**M**) Immunoblots for ubiquitinated proteins (Ubs), LC3B, and p62 in WT/KO cells over an LPS time course. (**N**) LC3B immunoblots in cells treated with LPS ± BAF. (**O**) Quantification of DALGreen signal (autolysosome indicator). (**P**) *Il1b* expression in cells treated with LPS alone or combined with CQ and/or CTM. (**Q**) Survival curves of septic mice treated with LPS ± CQ and/or CTM. (**R** and **S**) Immunoblots and quantification of ubiquitinated proteins in cells treated with LPS ± the proteasome inhibitor MG132. (**T**) Triple-IF for DAPI, proteasome subunit Psma5 (green), and Proteostat (red) in LPS-treated WT and KO cells. Data are presented as means ± SD. Statistical significance was determined by Student’s *t* test or log-rank test (Q); *P < 0.05, **P < 0.01, ***P < 0.001, ****P < 0.0001 compared with the LPS group (P and Q) or with the indicated group in other panels; #*P* < 0.05, ##*P* < 0.01 compared with the LPS + CQ + CTM group (P) or with either the LPS + CTM or LPS + CQ group (Q); ns, not significant. A.U., arbitrary units.

To investigate the functional role of Vim in LPS-mediated macrophage inflammation, we generated Vim-deficient (Vim-KO) RAW264.7 cells using CRISPR-Cas9 and performed RNA-seq analysis (fig. S4A and data S7). Principal components analysis (PCA) revealed distinct transcriptional profiles between WT and Vim-KO macrophages under basal conditions and following LPS stimulation (Fig. 4A). GSEA demonstrated significant down-regulation of the “Inflammatory Response” pathway in Vim-KO cells compared to WT cells upon LPS treatment (Fig. 4B). We subsequently identified DEGs with attenuated or enhanced responses to LPS in Vim-KO compared to WT cells (Fig. 4C). GO enrichment analysis of these DEGs revealed enrichment in key inflammatory pathways, including the receptor complex and plasma membrane signaling receptor complex (cellular component, CC), chemokine activity, cytokine receptor binding (molecular function, MF), and cytokine-mediated signaling pathway (biological process, BP), suggesting that Vim deficiency disrupts critical inflammatory signaling cascades (Fig. 4D). To further dissect transcriptional regulation, we analyzed TF activity using the network-based TF activity inference algorithm VIPER (*38*). Vim-KO macrophages exhibited significantly reduced activity of inflammation-associated TFs, including Foxm1, Stat6, Nfkb2, Fosl2, E2f4, Stat3, and Rel compared to WT cells (Fig. 4E). Conversely, TFs such as Ppara, Zkscan1, and Stat2 showed increased activity in Vim-KO cells, suggesting a potential compensatory transcriptional reprogramming in the absence of Vim (Fig. 4E). To validate the impaired inflammatory response, we measured cytokine expression using PCR analysis. Vim-KO cells exhibited significantly reduced LPS-induced expression of *Il1b* and *Il6* (Fig. 4F). Similarly, transient knockdown of Vim using small interfering RNA (siRNA) (fig. S4B) led to a marked suppression of *Il6* and *Tnf* expression in LPS-stimulated RAW264.7 cells (Fig. 4G), highlighting the therapeutic potential of targeting Vim. In addition, Vim-deficient RAW264.7 macrophages exhibited significantly reduced apoptosis following LPS stimulation, as indicated by decreased levels of cleaved caspase-3 (fig. S4C) and fewer apoptotic cell fragments (fig. S4D), together with enhanced clearance capacity in a hepatocyte coculture system (fig. S4, E and F). Collectively, these data suggest that Vim contributes to the regulation of macrophage inflammatory responses, apoptotic susceptibility, and clearance function following LPS stimulation.

### Vim remodeling regulates proteostasis in activated macrophages

Given that Vim is a cytoskeletal protein essential for maintaining cellular architecture and signal transduction, we hypothesized that its structural organization modulates macrophage inflammatory responses. Immunofluorescence analysis revealed that unstimulated macrophages displayed a typical elongated filamentous network radiating from the nucleus (Fig. 4H). Upon LPS stimulation, Vim expression was markedly up-regulated in both RAW264.7 cells (Fig. 4H) and mouse liver macrophages (fig. S3, B and C), which was accompanied by a morphological transition from thin filaments to thickened, curved, and cage-like structures (Fig. 4H). This reorganization was partially attenuated by CTM treatment (Fig. 4H) and was partially attenuated in TG2-KO cells (fig. S4G). Quantitative analysis confirmed a significant increase in the Vim-positive area after LPS treatment, which was partially reversed by CTM in RAW264.7 cells (Fig. 4I). Live-cell imaging further revealed Vim relocalization toward the nucleus, forming a perinuclear cage–like structure in response to LPS (Fig. 4J). Given the association between Vim cages and the sequestration of misfolded protein AGGs, we examined the spatial relationship between Vim and protein AGGs using ProteoStat staining combined with Vim immunofluorescence. In both LPS-treated RAW264.7 (Fig. 4K) and mouse liver tissues (Fig. 4L), Vim formed cage-like structures that closely surrounded protein AGGs, suggesting that Vim reorganization contributes to proteostasis by sequestering misfolded proteins. Consistent with this, Western blot analysis using anti-ubiquitin antibodies showed that Vim-KO cells exhibited reduced accumulation of ubiquitinated proteins at up to 12 hours post-LPS compared to WT cells (Fig. 4M), indicating an altered handling of misfolded protein stress. Collectively, these findings highlight the importance of Vim remodeling in the spatial management of protein AGGs during inflammatory activation.

### Vim mediates proteasome-dependent proteostasis

To further dissect how Vim regulates proteostasis at the systemic level, we examined the dynamics of two major protein quality control pathways: autophagy and UPS. LC3B-II, a lipidated form of LC3B, is widely used as a marker for autophagosome formation (*39*). Twelve hours after LPS stimulation, Western blot analysis revealed that the increase in LC3B-II levels upon bafilomycin A1 (BAF) treatment was attenuated in LPS-treated WT and Vim-KO cells compared to that in the PBS-treated controls (Fig. 4N). Because BAF inhibits lysosomal acidification and blocks autophagosome-lysosome fusion, the lower LC3B-II accumulation in LPS-treated cells suggests impaired autophagic flux (fig. S4H), consistent with the results of prior reports of LPS-induced autophagy impairment (*40*, *41*). Accordingly, changes in autophagic flux were quantified by measuring the average integrated intensity of DALGreen, a fluorescent probe for acidic autolysosomes. To assess the effect of LPS, DALGreen signals after BAF treatment were compared with their corresponding untreated (−BAF) controls, with a smaller reduction indicating lower basal autophagic flux (fig. S4H). LPS treatment resulted in a significantly smaller decrease in DALGreen intensity upon BAF inhibition in both WT and Vim-KO cells (fig. S4I), indicating reduced autophagic flux. When DALGreen intensities after BAF inhibition were further normalized to their respective PBS controls, Vim-KO cells exhibited significantly higher normalized DALGreen levels than WT cells (Fig. 4O), indicating a more pronounced suppression of autophagy in the absence of Vim. On the basis of these findings, we hypothesized that autophagy may serve as a compensatory response to misfolded proteins and ubiquitin stress, potentially exacerbating inflammation. To test this hypothesis, we performed functional analyses using pharmacological inhibition of autophagy with chloroquine (CQ) or genetic ablation of Atg5, a gene essential for autophagosome formation. Both CQ treatment and Atg5 deficiency significantly suppressed LPS-induced macrophage activation, particularly when combined with CTM treatment (Fig. 4P) or in Vim-KO or TG2-KO backgrounds (fig. S4, J to M), suggesting that persistent or compensatory autophagy exacerbates inflammation under stress. Notably, the combined administration of CQ and CTM conferred superior protection compared to either treatment alone and completely prevented LPS-induced mortality in septic mice over 4 days (Fig. 4Q). These results highlight the therapeutic potential of dual-targeting autophagy and TG2 activity in sepsis. Given these findings, we next evaluated the role of the UPS, whereby E3 ligase–mediated polyubiquitination targets misfolded proteins for degradation by the 26S proteasome. Immunoblotting with anti-ubiquitin antibodies, combined with MG132-mediated proteasome inhibition, revealed increased accumulation of ubiquitinated proteins in LPS-treated Vim-KO cells compared to PBS-treated controls (Fig. 4, R and S). These findings suggest enhanced dependence on proteasome-mediated degradation in the absence of Vim. Consistent with these observations, Vim deficiency promoted proteasome recruitment to protein AGGs in LPS-stimulated macrophages (Fig. 4T and fig. S4M), suggesting adaptive spatial reorganization of the proteolytic machinery. Together, these results indicate that Vim plays a critical role in coordinating autophagy and UPS activity, and that its absence promotes a proteasome-dependent proteostasis program that may mitigate stress-induced protein aggregation in inflamed macrophages.

### Proteomic profiling of protein aggregation in Vim-deficient macrophages

**Fig. 5.**
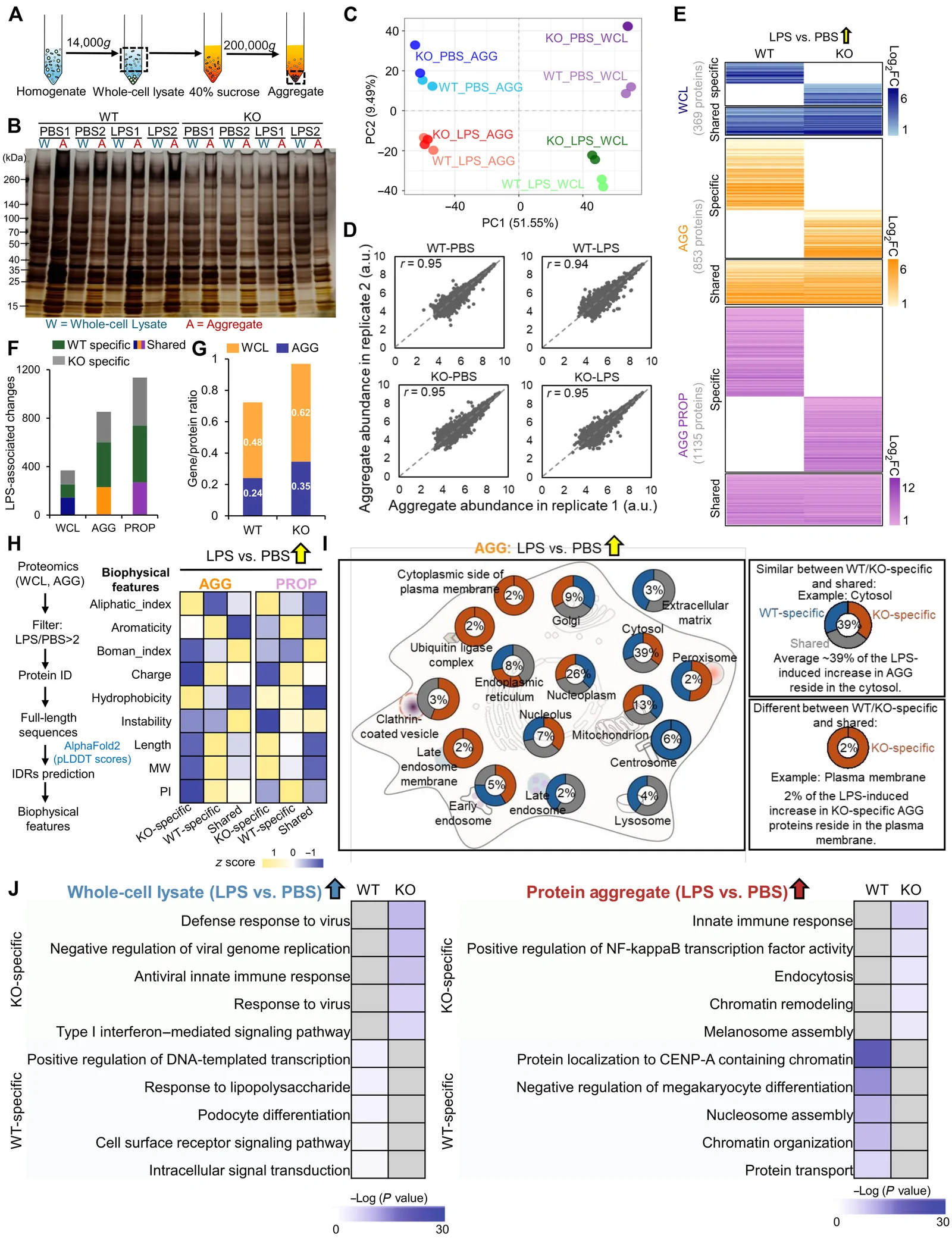
Proteomic profiling of protein aggregation in Vim-deficient macrophages. (**A**) Workflow for detergent-insoluble protein AGG isolation. (**B**) Silver-stained SDS-PAGE of WCL and AGG fractions isolated from WT and KO RAW264.7 cells treated with LPS (1 μg/ml) for 12 hours. (**C**) PCA of nLC-MS/MS–based proteomic profiles of WCL and AGG fractions. (**D**) Reproducibility of AGG proteomics. Pearson correlation (*r*) of log_10_-transformed protein abundance between replicates (a.u., arbitrary units). (**E**) Heatmap and (**F**) bar plot of up-regulated WCL, AGG, and AGG propensity (PROP) proteins. Proteins were categorized as genotype-specific (WT-specific or KO-specific) or shared between genotypes. (**G**) Overlap between LPS–up-regulated transcripts (RNA-seq) and proteins (nLC-MS/MS) in WCL and AGG fractions. (**H**) (Left) IDRs analysis strategy. pLDDT, predicted local distance difference test. (Right) Heatmap of normalized biophysical intensity of IDRs for up-regulated AGG and PROP proteins. (**I**) GO cellular component analysis of up-regulated AGG proteins. Arc lengths indicate category contributions; central values indicate average proportions. (**J**) GO biological process enrichment of up-regulated WCL and AGG proteins.

To characterize the molecular composition and regulation of LPS-induced protein AGGs, we adapted a serial centrifugation–based protocol to isolate detergent-insoluble AGGs under native conditions (Fig. 5A) (*42*). Whole-cell lysates (WCL) and AGG fractions were prepared from WT and Vim-KO RAW264.7 cells under basal and LPS-stimulated conditions (Fig. 5B). Enrichment of the AGG fraction was confirmed by the absence of soluble glyceraldehyde-3-phosphate dehydrogenase (GAPDH) and the retention of structurally stable β-actin (fig. S5A). Subsequent nLC-MS/MS–based proteomic analyses of the WCL and AGG fractions identified more than 6000 proteins (data S8). PCA revealed a clear separation based on genotype and treatment, with distinct clustering of the WCL and AGG fractions across conditions (Fig. 5C). Pairwise correlation analysis of biological replicates demonstrated high intragroup consistency in protein abundance measurements (Fig. 5D), confirming the robustness and reproducibility of the proteomic profiling approach. Proteomic profiling revealed divergent aggregation landscapes between WT and Vim-KO macrophages following LPS stimulation. While 369 proteins were significantly up-regulated in the WCL fraction, 853 proteins were enriched in the AGG fraction across the genotypes (Fig. 5E). Notably, the identity of the aggregated proteins was highly genotype-specific, with limited overlap between WT and Vim-KO cells (Fig. 5F). Although WT cells showed a greater overall accumulation of proteins in the AGG fraction, both genotypes exhibited similar numbers of proteins, with an increased aggregation propensity upon LPS stimulation. Transcriptomic-proteomic integration revealed limited transcriptional contribution to protein aggregation. In WT cells, only 24% of the AGG-enriched proteins corresponded to LPS–up-regulated genes, compared to 48% in the WCL fraction. Similarly, in Vim-KO cells, 35% of AGG proteins matched LPS-induced transcripts, whereas this proportion reached 62% in WCL (Fig. 5G). Correlation analysis further demonstrated weaker transcript-protein concordance in AGG fractions compared to WCL [correlation coefficient (*r*) = 0.485 ± 0.049 for WCL; *r* = 0.365 ± 0.049 for AGG; fig. S5B], indicating the predominant role of posttranscriptional mechanisms in driving protein aggregation under inflammatory stress.

### Vesicular trafficking alterations in Vim-deficient macrophages

To better understand the molecular properties driving protein aggregation, we analyzed the biochemical and biophysical characteristics of proteins enriched in the AGG fraction after LPS stimulation, with a specific focus on intrinsically disordered regions (IDRs), which are key motifs involved in protein-protein interactions (*43*). Aggregated proteins were categorized as WT-specific, Vim-KO-specific, or shared, and subsequently evaluated for nine IDR-related features using AlphaFold2-based predictions (Fig. 5H, left). In WT cells, aggregation was associated with increased positive charge, aromaticity, and isoelectric point, whereas in Vim-KO cells, aggregated proteins displayed stronger correlations with negative charge, hydrophobicity, and aliphatic index (Fig. 5H, right). These data suggest that Vim influences the biophysical environment governing aggregation propensity during inflammation. Subsequently, we investigated the subcellular localization of aggregated proteins in each group (Fig. 5I). Upon LPS stimulation, plasma membrane proteins, ubiquitin ligase complexes, and late endosomal membrane components were preferentially enriched in AGGs of Vim-KO cells, whereas centrosomal proteins were predominantly aggregated in WT cells. Notably, Vim-KO–specific AGGs were largely depleted of late endosomal and lysosomal compartments, whereas WT-specific AGGs lacked clathrin-coated vesicle components, indicating distinct genotype-dependent trafficking patterns. Functional enrichment analysis further revealed that shared AGGs were linked to canonical LPS response pathways (fig. S5C), whereas Vim-KO–specific AGGs were uniquely enriched in innate immune regulators, NF-κB activators, and endocytic machinery (Fig. 5J). Collectively, these findings demonstrate that Vim deficiency reshapes vesicular trafficking and AGG composition, thereby leading to altered proteostasis and inflammatory signaling in macrophages.

### Rab27a-positive vesicles facilitate proteasomal degradation of inflammatory AGGs

**Fig. 6.**
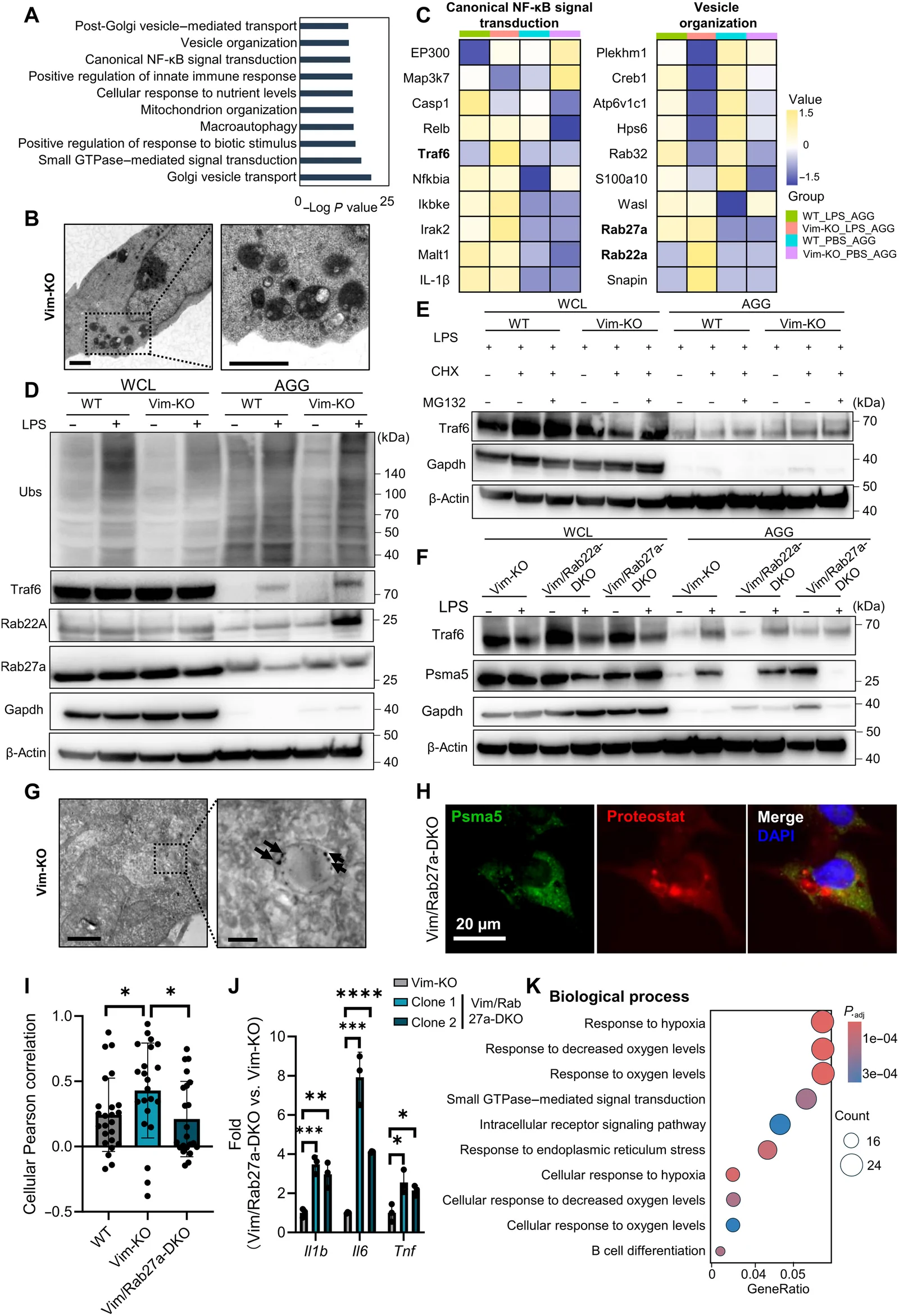
Rab27a-positive vesicles facilitate proteasomal degradation of inflammatory AGGs. (**A**) GO biological process enrichment of up-regulated AGG proteins comparing LPS-treated Vim-KO and WT RAW264.7 cells. (**B**) Transmission electron microscopy images of Vim-KO cells treated with LPS (1 μg/ml) for 12 hours. Right: magnified view. Scale bars, 1 μm. (**C**) Heatmap of protein abundance for “canonical NF-κB signaling” and “vesicle organization” GO terms. (**D**) Immunoblots of ubiquitinated proteins (Ubs), Traf6, Rab22a, and Rab27a in AGG and WCL fractions from LPS-treated WT and Vim-KO cells. (**E**) Immunoblots of Traf6 in WCL and AGG fractions from LPS-treated WT and Vim-KO cells ± CHX (last 45 min) or MG132 (last 15 min). (**F**) Immunoblots of Traf6 and Psma5 in WCL and AGG fractions from LPS-treated Vim-KO, Vim/Rab22a-DKO, and Vim/Rab27a-DKO cells. (**G**) Immunogold electron microscopy of LPS-stimulated Vim-KO cells labeled with an anti-Rab27a antibody. Black arrows indicate Rab27a-positive structures. Scale bars, 500 nm (main panel) and 100 nm (inset). (**H**) Immunofluorescence staining of DAPI (blue), Psma5 (green), and Proteostat (red) in LPS-treated Vim/Rab27a-DKO cells. (**I**) Quantification of intracellular colocalization between Psma5 and protein AGGs, expressed as Pearson’s *r* calculated from pixel-wise fluorescence intensities. (**J**) Relative gene expression of *Il1b*, *Il6*, and *Tnf* in LPS-treated Vim-KO and two independent Vim/Rab27a-DKO clones. (**K**) Bubble plot of top 10 enriched GO biological processes of proteins uniquely up-regulated in Vim/Rab27a-DKO cells following LPS stimulation. Data are presented as means ± SD; **P < 0.01, ***P < 0.001, Student’s *t* test or Kruskal-Wallis test.

Building on the observation that Vim deficiency reshapes the aggregation landscape and alters vesicular trafficking, we examined how this reorganization influences the degradation of inflammatory mediators. Given the enrichment of NF-κB regulators and ubiquitinated proteins in Vim-KO AGGs, we hypothesized that their sequestration into discrete compartments facilitates targeted proteasomal degradation, thereby attenuating inflammatory signaling during proteotoxic stress. In support of this hypothesis, GO enrichment analysis of the Vim-KO AGG fraction revealed significant enrichment of pathways related to innate immune response, canonical NF-κB signaling, and vesicle organization (Fig. 6A). Electron microscopy revealed clusters of endosome-like vesicles near the plasma membrane in LPS-treated Vim-KO and TG2-KO cells but not in WT cells (Fig. 6B and fig. S6A). These observations prompted further investigation of the association between vesicle-related regulators and inflammatory pathways. Heatmap analysis demonstrated that key regulators of canonical NF-κB signaling and vesicle organization, including Traf6, Rab22a, and Rab27a, were selectively enriched in the AGG fraction of Vim-KO cells (Fig. 6C). Western blot analysis further confirmed elevated levels of these proteins in Vim-KO AGGs compared with WT AGGs under LPS stimulation (Fig. 6D and fig. S6B). Consistent with previous findings, Vim deficiency reduced the abundance of ubiquitinated proteins in the WCL while increasing their accumulation in the AGG fraction (Fig. 6D). This finding suggests the sequestration of ubiquitinated proteins, such as Traf6, into AGGs. Given our earlier observation that Vim-KO AGGs were enriched in proteasome components, we next investigated whether Traf6-containing AGGs were subject to proteasomal degradation. We treated cells with the translation inhibitor cycloheximide (CHX). In WT cells, CHX reduced Traf6 levels in the AGG fraction, suggesting that AGG formation relied on newly synthesized Traf6 (Fig. 6E). Conversely, CHX-treated Vim-KO cells showed an increased accumulation of Traf6 in the AGG, indicating that preexisting Traf6 had a higher aggregation propensity in the absence of Vim. Treatment with the proteasome inhibitor MG132 led to further Traf6 accumulation in the AGG fraction of Vim-KO cells relative to that in WT cells, suggesting enhanced proteasome-mediated clearance of Traf6 AGGs under Vim-deficient conditions (Fig. 6E). Because RAB guanosine triphosphatases (GTPases) regulate vesicle trafficking and cargo sorting (*44*, *45*), we next investigated whether Rab22a or Rab27a contributes to the formation or turnover of inflammatory AGGs. To examine this, we generated Vim/Rab22a and Vim/Rab27a double KO (DKO) cells (fig. S6, C and D). The LPS-induced accumulation of Traf6 in AGG fractions was significantly impaired by Rab27a deletion and moderately reduced by Rab22a deficiency (Fig. 6F and fig. S6E), suggesting that Rab22a and Rab27a are required for the aggregation or trafficking of Traf6. Consistent with this, immuno-electron microscopy revealed Rab27a localization to vesicle-like structures in the cytoplasm of LPS-stimulated Vim-KO cells (Fig. 6G). In contrast, the nonaggregated pool of Traf6 was abundant under basal conditions and showed a reduction following LPS stimulation in both Vim-KO and DKO cells (Fig. 6F). Consistently, time-course analysis in WT macrophages revealed a decrease in total Traf6 protein as early as 30 min after LPS stimulation (fig. S6F). These data indicate that changes in the composition of aggregated assemblies correlate with LPS-induced inflammatory responses more closely than changes in the soluble Traf6 pool. In line with this, Rab27a deficiency significantly disrupted the recruitment of proteasome components to AGGs (Fig. 6, F, H, and I), suggesting an association between aggregation and proteasome recruitment. Functionally, Rab27a deletion partially restored LPS-induced expression of *Il1b*, *Il6*, and *Tnf* (Fig. 6I), as well as apoptotic responses (fig. S6G), in Vim-KO cells. To systematically characterize the transcriptional programs associated with Rab27a deficiency in LPS-stimulated Vim-KO macrophages, we performed RNA-seq analysis. PCA revealed that LPS treatment and Rab27a status accounted for most transcriptomic variance along PC1 and PC2, respectively (fig. S6I). Upon LPS stimulation, 492 genes were uniquely up-regulated in Vim/Rab-DKO RAW264.7 cells compared with Vim-KO cells (fig. S6K). GSEA indicated that these genes were enriched in pathways related to hypoxia and endoplasmic reticulum (ER) stress (Fig. 6J). Together, these findings support a model in which Vim deficiency promotes Rab27a-dependent sequestration of Traf6 into proteasome-accessible AGGs under inflammatory stress, accompanied by altered stress-associated transcriptional and apoptotic responses in Vim-KO macrophages.

## DISCUSSION

**Fig. 7.**
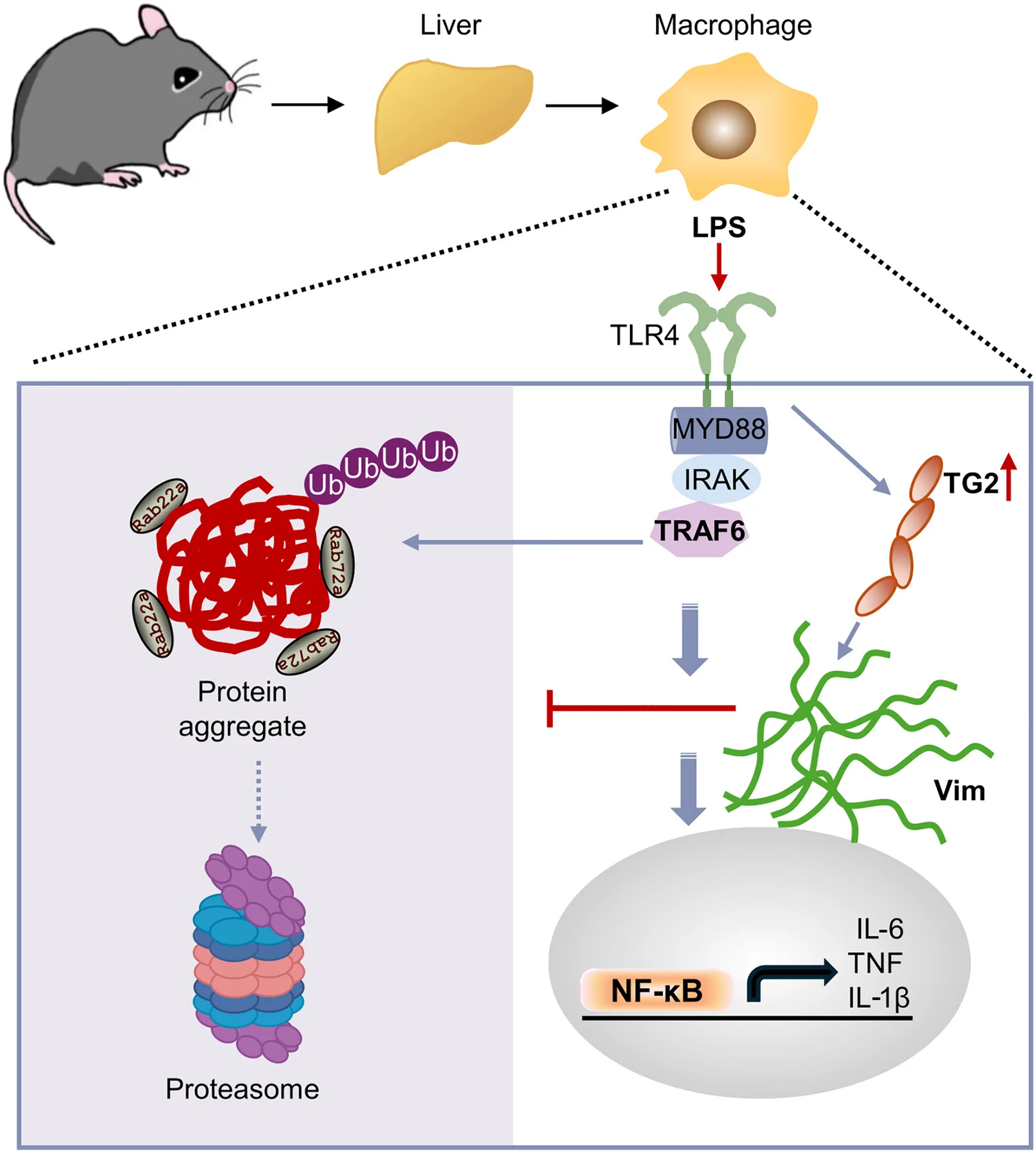
Model of proteostasis-dependent TG2-Vim axis in macrophage activation. LPS triggers TG2 transamidase activity in hepatic macrophages. Pharmacological or genetic TG2 inhibition improves sepsis survival and attenuates inflammation. TG2 cross-links Vim, whose deletion similarly reduces inflammation. Vim deficiency promotes proteasomal degradation of TRAF6 within Rab22a/Rab27a-regulated AGGs.

Sepsis, particularly SALD, remains a major clinical challenge with limited therapeutic options targeting the inflammatory cascade. Our study uncovered a previously unrecognized mechanism driving SALD pathogenesis: TG2-mediated Vim cross-linking disrupts liver macrophage proteostasis, amplifies inflammatory signaling, and ultimately exacerbates liver injury (Fig. 7). By bridging cytoskeletal dynamics, protein aggregation, and vesicle trafficking, our findings reframe SALD as a disorder of proteostasis, not solely a cytokine dysregulation, thus offering a conceptual basis for understanding the pathological mechanisms of SALD.

TG2 has been well documented to promote inflammation across multiple inflammatory contexts, including neuroinflammation and chronic inflammatory diseases [reviewed in (*13*)]. Early studies demonstrated increased total tissue transglutaminase activity in bulk liver tissue following LPS administration, with an emphasis on extracellular activity and matrix-associated functions, particularly in fibroblasts, rather than intracellular enzymatic activity (*46*). However, the role of TG2 in inflammation and sepsis remains controversial. Genetic deletion of TG2 has been reported to improve survival in LPS-induced septic shock, accompanied by a marked reduction in inflammatory responses and attenuated organ damage, partly through regulation of dendritic cell differentiation (*47*, *48*). In contrast, in TNF-α–dependent septic models, TG2 exhibited a protective role in liver injury (*49*). TG-mediated cross-linking of drosocrystallin fibers in the *Drosophila* gut also plays a protective role to form a physical barrier against exotoxins from invading pathogenic microbes (*50*). Collectively, these opposing effects of TG2 are likely determined by cell type, disease stage, and genetic background, such as inflammatory stress in immune cells versus apoptotic stress in epithelial cells. The protective role of TG2 has been attributed to a nonenzymatic function mediated by its N-terminal domain, which facilitates cargo recognition and autophagic degradation independently of transamidase activity (*51*). In contrast, our study directly visualizes and functionally interrogates early-stage intracellular TG2 transamidase activity in liver macrophages during endotoxemia. Using unbiased biotinylated substrate proteomics, we identify Vim as a direct substrate of TG2-mediated cross-linking, rather than a TG2-binding protein, and demonstrate that TG2-Vim cross-linking promotes cytoskeletal remodeling, disrupts proteostasis, enhances inflammatory responses, increases apoptosis, and impairs macrophage clearance capacity. These findings define a mechanistically distinct enzymatic role of TG2 in acute inflammation. Notably, both our study and prior work converge on proteostasis as a central determinant of TG2 function, suggesting that differential engagement of enzymatic versus nonenzymatic TG2 activities may underlie its context-dependent roles in inflammation and sepsis.

Vim has been well-studied in bacterial infections because of its role in vacuole formation and intracellular trafficking (*52*, *53*). We expanded its function to include proteostatic regulation under inflammatory stress. As a key cytoskeletal element, Vim maintains structural integrity and acts as a stress-responsive scaffold that orchestrates various physiological and pathological processes (*54*). For example, it anchors the ER quality control compartment near endolysosomes to support ER stress resolution (*55*) and coordinates proteasome recruitment to protein AGGs in adult neural stem cells to maintain proteostasis (*56*). However, in LPS-activated macrophages, Vim filaments reorganize into cage-like structures, which may restrict proteasomal access to protein AGGs. While protein aggregation typically serves as a cytoprotective role by sequestering misfolded proteins (*27*), we found that Vim deficiency uniquely reshapes this process by altering AGGs composition rather than increasing overall aggregation. Specifically, Vim-deficient macrophages form AGGs that resemble early endosomal compartments enriched in the ubiquitination machinery, membrane trafficking complexes, and inflammatory mediators, which are distinct from the classical endosome-to-lysosome degradation pathway. Accordingly, Vim deficiency altered the composition of LPS-induced AGGs, leading to increased accumulation of proteins involved in the positive regulation of NF-κB signaling. Among these, Traf6, a key adaptor in the MyD88-dependent branch of TLR4 signaling (*57*), was markedly enriched in AGGs from Vim-deficient cells. These AGGs also exhibited elevated ubiquitination, likely enhancing proteasome recruitment and accelerating Traf6 degradation in the absence of Vim. The differential centrifugation–based AGGs isolation protocol used in this study physically separates insoluble, high–molecular weight protein assemblies from the soluble proteome, large macromolecular complexes, ribonucleoprotein particles, subcellular organelles, and large biomolecular condensates (*42*). Traf6 has been shown to undergo liquid-liquid phase separation during early LPS signaling to enable oligomerization and NF-κB activation (*58*). Consistent with this, our data reveal a rapid shift of Traf6 from the soluble pool toward detergent-insoluble, aggregated assemblies following LPS stimulation. These findings indicate that Traf6 signaling competence is dictated by its biophysical state and AGGs composition, such as proteasome enrichment, rather than total protein abundance in shaping downstream inflammatory responses.

Protein AGGs can be degraded through either the proteasomal or lysosomal pathway. Our proteomic analysis revealed that WT macrophages preferentially directed AGGs toward the endocytic-lysosomal route, as evidenced by the enrichment of late endosomal and lysosomal proteins. This canonical degradation pathway relies on microtubule-based transport toward the perinuclear region, which may explain the absence of vesicle clusters near the plasma membrane in WT cells. In contrast, Vim-deficient cells lack this lysosomal signature and instead accumulate proteasome-associated proteins in their AGGs, indicating a compensatory shift toward proteasome-mediated clearance. This divergence in trafficking may stem from the structural rearrangement of Vim filaments upon LPS stimulation, which form cage-like structures that encircle or overlap with AGGs in WT cells. Such structures have previously been reported to enclose centrosome-associated proteins (*53*), which is consistent with our finding that WT AGGs were enriched in centrosomal components. Together, these results indicate that Vim not only shapes the physical architecture of AGGs but also dictates their subcellular routing and degradation fate, thereby affecting the inflammatory output of macrophages during sepsis.

A key mechanistic insight is that Rab GTPases, especially Rab27a, serve as critical regulators that link vesicle trafficking to proteasome-mediated degradation of inflammatory AGGs. Rab GTPases are localized in specific intracellular membranes, where they orchestrate vesicle budding, transport, and fusion by recruiting distinct sets of effector proteins (*44*, *45*). This spatial and temporal coordination of intracellular trafficking is essential for immune cell function and rapid inflammatory response mobilization (*59*, *60*). Besides their general trafficking roles, Rab GTPases modulate immune signaling by controlling receptor localization and facilitating endocytic and phagocytic processes (*44*, *61*). Rab27a, for example, is known to regulate secretory pathways in immune cells, including cytokine release, such as TNF, during systemic inflammation (*62*). The specificity and outcome of Rab GTPase–mediated trafficking are determined by the repertoire of Rab effectors expressed by each cell type (*63*). In our study, Vim deficiency in macrophages resulted in increased accumulation of Rab27a within LPS-induced protein AGGs, implicating these GTPases in the intracellular routing of Traf6. This enrichment may reflect altered Rab effector recruitment or trafficking behavior in the absence of Vim scaffolding. Notably, Rab27a deficiency selectively impaired proteasome recruitment to Traf6-containing AGGs, suggesting a role for Rab27a in facilitating vesicle-proteasome docking. Previous studies have reported that Rab27a-deficient mice show improved survival and reduced hepatic neutrophil infiltration following LPS challenge (*62*). Together, these findings highlight Rab27a as a context-dependent regulator of inflammation and suggest that it may function at the interface between trafficking machinery and proteasomal clearance to resolve proteotoxic stress in macrophages.

In summary, our findings suggest that the TG2-Vim axis represents a mechanistic link between cytoskeletal remodeling and regulation of proteostasis during sepsis-induced inflammation. The anti-inflammatory effects of cysteamine, the reduced monomeric form of CTM, have been well documented in diverse disease contexts, including skin inflammation, diabetic retinopathy, cystic fibrosis, and COVID-19 (*64*–*67*). Notably, the synergistic survival benefit observed with combined CTM and autophagy inhibitor CQ treatment further supports the translational potential of targeting TG2-dependent proteostasis pathways in sepsis. Nevertheless, some limitations remain as this study relied on an acute LPS challenge model and murine macrophage cell lines, which may not fully recapitulate the complexity of polymicrobial or viral sepsis in human patients. Future investigations using human liver macrophages and clinically relevant sepsis models are essential to validate TG2 as a biomarker and to advance therapeutic strategies targeting the TG2-Vim-proteostasis axis in SALD.

## MATERIALS AND METHODS

### Clinical samples

Human liver tissues were obtained from the Liver and Gallbladder Research Center and the intensive care unit of Drum Tower Hospital (Nanjing, China). The study cohort comprised 7 patients diagnosed with SALD and 14 control patients with noninflammatory hepatic conditions (hemangioma, hepatocellular carcinoma, or hepatolithiasis). During surgical procedures or biopsy, tissue samples were collected, immediately snap-frozen in liquid nitrogen, and stored at −80°C until analysis. The study protocol was approved by the Ethics Committee of the Affiliated Drum Tower Hospital, Medical School of Nanjing University (approval no. 2023-396-03). All patients provided written informed consent before participation in the study.

### Animal experiment

Male C57BL/6J mice (6 to 8 weeks or 16 months old) were purchased from CLEA Japan Inc. (Tokyo, Japan). The mice were housed under constant temperature (22° ± 1°C) with ad libitum access to food and water. TG2-KO mice were generated as described previously (*68*). For each experiment, mice were randomly assigned to experimental groups. LPS (10 or 30 mg/kg), CTM (50 or 100 mg/kg), and CQ (100 mg/kg; Sigma-Aldrich) were administered via intraperitoneal (ip) injection. All procedures were approved by the Institutional Committee of Animal Experiments of RIKEN (AEY2022-032) and conducted in accordance with the ARRIVE guidelines (*69*) and the institutional and national guidelines for the care and use of laboratory animals.

### Macrophage depletion and reconstitution in vivo

Macrophages were depleted using clodronate liposomes, as previously described (*70*). Briefly, male C57BL/6J mice (6 to 8 weeks old) received a single intraperitoneal injection of clodronate liposomes (Hygieia Bioscience, #16004473) or control PBS-liposomes (200 μl/20 g body weight) 24 hours before LPS administration (10 mg/kg, ip). The depletion efficiency was assessed by F4/80 immunostaining or by quantitative PCR analysis of *Adgre1* expression in tissue samples. For macrophage reconstitution, WT or Vim-KO RAW264.7 cells (2 × 10^6^ cells per mouse) were intravenously injected 24 hours after clodronate liposome treatment, as previously described (*33*). LPS (10 mg/kg, ip) was administered the following day, and liver tissues were collected 6 hours after LPS injection. To validate macrophage reconstitution, RAW264.7 cells were labeled with the fluorescent lipophilic dye CM-DiI before injection and subsequently detected by coimmunostaining with F4/80 in liver sections.

### Cell culture and stimulation

Human hepatic Hc cells (Cell Systems) and mouse RAW264.7 macrophage-like cells (RIKEN Cell Bank) were cultured in Dulbecco’s modified Eagle’s medium (Gibco) or minimum essential medium (MEM, Nacalai Tesque) supplemented with 10% fetal bovine serum and 1% penicillin-streptomycin. RAW264.7 cells were additionally supplemented with 0.1 mM nonessential amino acids. To generate a CM, RAW264.7 cells were cultured in phenol red–free, serum-free MEM (Gibco) and stimulated with LPS (1 μg/ml) in the presence or absence of CTM (0.5 mM) or NC9 (50 μM). MG132 (10 μM), CHX (1 mM), or BAF (100 nM) were added at defined intervals before harvest. All cells were maintained at 37°C in 5% CO_2_.

### Plasmid construction and transfection

The mCherry-tagged full-length Vim, HypBase transposase, and piggyBac mCherry– or EGFP-labeled CRISPR-Cas9 plasmids were synthesized using VectorBuilder (Yokohama, Japan). EGFP-tagged full-length TG2 was cloned into the pEGFP-C1 vector (Clontech) from the TG2-pSG5 construct, as previously described (*71*). Transfection was carried out using Lipofectamine 3000 with the P3000 reagent (Thermo Fisher Scientific) following the manufacturer’s instructions. For CRISPR-Cas9–mediated KO, RAW264.7 cells were transfected with plasmids encoding gene-specific guide RNAs (gRNAs) in combination with the HypBase transposase at a molar ratio of 2:1. The gRNA target sequences were as follows: Vim, CGGGTCACATAGGCGCCACC; Tgm2, TCCCATCTATGTGGGCCGCG; Atg5, TTCCATGAGTTTCCGGTTGA; Rab22a, AGCATTGTCGTTGCCATCGC; and Rab27a, GTTTCCTCAATGTCCGAAAC. Single cells were sorted on the basis of mCherry or EGFP fluorescence using a BD FACSAria II into 96-well plates and expanded under neomycin or puromycin selection. The clonal cell lines were validated by Western blotting to confirm the loss of target protein expression.

### siRNA transfection

Cells were transfected with 200 nM siRNA-targeting Vim (sc-29523) or control siRNA (sc-37007, Santa Cruz Biotechnology) using Lipofectamine 3000 according to the manufacturer’s protocol.

### Live cell imaging

Cells were seeded in 24- or 96-well plates and maintained at 37°C in 5% CO_2_. Time-lapse imaging was performed using the Incucyte Live-Cell Analysis System (Sartorius) at 10× or 20× magnification with hourly acquisition over 24 hours in the bright-field and fluorescent channels (green/red).

### Autophagic flux assay

Autophagic flux was assessed using the Autophagic Flux Assay Kit (#A562, Dojindo). Cells were incubated with 1 μM DALGreen and 0.2 μM DAPRed for 30 min at 37°C, washed twice, and then treated with PBS or LPS, with or without BAF. DALGreen fluorescence was quantified using the Incucyte system.

### Western blotting

Western blotting was performed as previously described (*72*). For denaturing conditions, cells were lysed using M-PER^TM^ protein extraction reagent (Thermo Fisher Scientific). The denatured protein samples were separated by SDS-PAGE and transferred to polyvinylidene difluoride (PVDF) membranes. For nondenaturing conditions, cells were lysed in NativePAGE sample buffer (Thermo Fisher Scientific) containing 10% *n*-dodecyl-β-d-maltoside and subjected to electrophoresis using a nondenaturing gel (8 to 16% Mini-Protean TGX gel, Bio-Rad Laboratories) and then transferred to PVDF membranes. The membranes were incubated overnight at 4°C with the following primary antibodies at the indicated dilutions: anti-TG2 (1:1000), anti-Vim (1:500, Cell Signaling Technology 5741S or Santa Cruz Biotechnology sc-373717), anti-ubiquitin (1:1000, Santa Cruz Biotechnology sc-8017), anti-LC3B (1:1000, Cell Signaling Technology 2775S), anti-p62 (1:1000, Santa Cruz Biotechnology sc-48402), anti-Traf6 (1:1000, Cell Signaling Technology 67591 T), anti-Rab22a (1:1000, Proteintech 12125-1-AP), anti-Rab27a (1:1000, Cell Signaling Technology 69295T), anti-α5 subunit of the proteasome (Psma5; 1:1000, Abcam ab189855), anti-caspase 3 (Casp3; 1:1000, Biovision 3004-100), anti–cleaved caspase-3 (clCasp3; 1:1000, Cell Signaling Technology, 9661), anti-GAPDH (1:1000, BioLegend 607902), and anti–β-actin (1:1000, Cell Signaling Technology, 3700). The next day, the membranes were washed three times with Tris-buffered saline containing 0.1% Tween-20 (TBST) and incubated with HRP-conjugated secondary antibodies (1:5000 dilution). Signals were detected using ECL Prime Western Blotting Detection Reagent (Cytiva, Marlborough, MA, USA). Data were quantified with ImageJ software.

### Real-time PCR

Total RNA was extracted, quantified, and reverse transcribed into cDNA as described previously (*72*). PCR was performed using TB Green Premix Ex Taq II (TaKaRa Bio) on a StepOne Real-Time PCR System (Applied Biosystems). The primer sequences used for amplification are listed in table S1.

### Enzyme-linked immunosorbent assay

Serum cytokine production was measured by enzyme-linked immunosorbent assay of TNF-α (BioLegend, 430904) and IL-6 (BioLegend, 431304) according to the protocol of the manufacturer.

### Determination of intracellular ROS production

RAW264.7 cells were seeded in 96-well plates and incubated overnight at 37°C with 5% CO_2_. The next day, the cells were treated with LPS (1 μg/ml) for 12 hours. Intracellular ROS levels was analyzed using the fluorescent probe CM-H_2_DCFDA (Life Technologies) at a final concentration of 2.5 μM. After incubation with the probe for 30 min at 37°C, fluorescence signals were measured using ApoTome laser scanning confocal microscope (Carl Zeiss Inc.). For each well, five to nine randomly selected fields of view were captured, and fluorescence intensity was quantified using the EBImage R packages.

### Evaluation of macrophage-mediated clearance activity

Macrophage-mediated clearance was evaluated by adapted from a previously described protocol (*73*). The fluorescent lipophilic dye CM-DiI–labeled RAW264.7 cells was stimulated with LPS (1 μg/ml) for 4 hours and then cocultured with GFP-expressing human hepatocyte cell line Hc for 2 hours. Phase contrast, GFP (488 nm), and CM-DiI (555 nm) images were automatically acquired after washing (0 hours) and after 24 hours of coculture using incuCyte system. Clearance burden index (CBI) was developed to objectively quantify macrophage-mediated clearance activity. For each imaging field, the CBI was defined as the total area of CM-DiI–positive macrophages divided by the total area of intact GFP-positive hepatocytes. The change in macrophage-mediated clearance activity over time was expressed as ΔCBI, calculated by subtracting the CBI at 0 hours from the CBI at 24 hours for the same field.

### Immunostaining and Proteostat staining

For tissue sections, mouse liver, kidney, and lung samples were embedded in optimum cutting temperature compound, snap-frozen, and sectioned at 10-μm thickness. Cryosections and cultured cells were fixed in 4% paraformaldehyde (PFA) for 10 min at room temperature (RT), followed by permeabilization in PBS containing 0.3% Triton X-100 for 10 min at RT. Blocking was performed in PBS containing 0.3% Triton X-100 and 5% normal donkey serum for 1 hour at RT. Sections and cells were then incubated overnight at 4°C with primary antibodies, including anti-F4/80 (1:200, Abcam, ab6640), anti-TG2 (1:200), anti-Vim (1:200, Cell Signaling Technology, 5741S; Santa Cruz Biotechnology, sc-373717), anti–cleaved caspase-3 (clCasp3; 1:400, Cell Signaling Technology, 9661), and anti-Psma5 (1:200, Abcam ab189855). The following day, the samples were washed three times for 10 min each with PBS or PBS containing 0.05% Tween-20 (PBST) and incubated for 1 hour at RT with secondary antibodies (1:500) and 4′,6-diamidino-2-phenylindole (DAPI, 1:2000) diluted in blocking buffer. In experiments assessing protein AGGs, Proteostat dye (1:2000, Enzo Life Sciences) was included with the secondary antibodies and DAPI. For tissue samples, sections were rinsed with PBST and mounted using Fluoromount (Diagnostic Biosystems, K024-200). To evaluate the colocalization between Psma5 and protein AGGs, images were automatically acquired using incuCyte system. Cells were classified as double-positive on the basis of global intensity thresholds derived from the entire dataset. Colocalization between the two channels was assessed at the single-cell level by calculating Pearson’s *r* across all intracellular pixels, with higher values indicating stronger spatial overlap between Psma5 and AGG signals.

### TG2 substrate identification

To identify TG2 substrates, cells were incubated with 1.5 mM 5BAPA at 37°C for 1 hour to covalently label TG2 substrates. Following incubation, cells were lysed using M-PER^TM^ Mammalian Protein Extraction Reagent (Thermo Fisher Scientific). Total protein concentration was determined using the BCA assay. Equal amounts of protein lysates were incubated with streptavidin-conjugated magnetic beads (Tamagawa Seiki) at 4°C for 1 hour with rotation. The beads were washed three times with 100 mM KCl buffer and separated using a magnetic stand. Bound proteins were eluted by boiling in 1× SDS sample buffer at 98°C for 5 min. The eluates were analyzed by SDS-PAGE followed by silver staining and/or subjected to nLC-MS/MS–based proteomic analysis.

### Isolation of WCL and AGG fractions from cultured cells

Cells were lysed in buffer A [30 mM tris-HCl (pH 7.5), 1 mM dithiothreitol (DTT), 40 mM NaCl, 3 mM CaCl_2_, 3 mM MgCl_2_, 5% glycerol, 1% Triton X-100, and protease inhibitor cocktail used at 1×] and centrifuged at 800*g* for 10 min to remove debris. Supernatants were collected and treated with ribonuclease A (100 μg/ml) and deoxyribonuclease I (100 μg/ml) for 30 min on ice. The samples were centrifuged at 14,000*g* for 15 min to obtain the WCL fraction. An aliquot of the WCL was reserved for protein quantification and further analysis. For AGG isolation, the remaining WCL was layered onto a 40% sucrose pad in a Beckman Coulter ultracentrifuge tube (catalog no. 358120). Ultracentrifugation was performed at 200,000*g* for 1 hour (Beckman SW 40 Ti rotor). The supernatant was removed, leaving approximately 20 μl of liquid at the bottom. The pellet was resuspended in 30 μl of buffer A. Protein concentrations of WCL and AGG fractions were measured using a BCA assay (TaKaRa BCA Protein Assay Kit, catalog no. T9300A).

### Silver staining

Equal amounts of WCL and AGG fractions or streptavidin bead pull-down protein lysates were resuspended in fivefold SDS sample buffer and resolved on 5 to 20% polyacrylamide gradient gels. Silver staining was performed using a commercial silver staining kit (Nacalai Tesque, 05773-11). The gels were fixed in a fixing solution for 10 to 20 min at RT. Following two washes in water for 10 min each, the gels were incubated in staining solution for 5 to 30 min. Once the desired band intensity was achieved, the reaction was quenched by adding 1% acetic acid to the solution. The gels were thoroughly rinsed with water to remove any residual reagents. Silver-stained gels were imaged using a Fusion System (Vilber Lourmat).

### RNA sequencing

For RNA-seq, total RNA was extracted using a FastGene RNA Basic Kit (NIPPON Genetics) and quantified using a NanoDrop spectrophotometer (Thermo Fisher Scientific). RNA-seq libraries were prepared using a TruSeq Stranded mRNA Library Prep Kit (Illumina) and sequenced on a DNBSEQ-G400 (MGI, Shenzhen, China) or Illumina NovaSeq 6000 platform (Illumina) in paired-end mode. Reads were aligned to the UCSC mm10 (GRCm38) mouse reference genome using salmon, and transcript abundance was quantified as transcripts per million. Differential gene expression, PCA, and GO enrichment analyses were performed using R (v4.0.3). Moreover, RNA-seq data were analyzed and visualized using MetaboAnalyst (https://metaboanalyst.ca/) and R (v 4.3.1).

### Fluorescence-activated cell sorting

Flow cytometry analysis and cell sorting were performed as described previously (*74*). Following labeling with 5BAPA, the cells were stained with TRITC-conjugated streptavidin (1:250) at RT for 1 hour. Cell sorting was performed using a BD FACSAria flow cytometer (BD Biosciences, San Diego, CA, USA). Data were analyzed using FlowJo software (TreeStar).

### Proximity ligation assay

PLA was performed using Sigma-Aldrich’s Duolink in situ PLA system, including the anti-rabbit PLUS probe (DUO92002-30RXN), anti-mouse MINUS probe (DUO92004-30RXN), and red detection reagents (DUO92008-30RXN), following the manufacturer’s instructions. Cells were fixed and permeabilized as described in the immunostaining section and incubated overnight at 4°C with primary antibodies, including rabbit anti-TG2 and mouse anti-Vim. The following day, the cells were washed twice for 5 min with buffer A. PLA probes (PLUS and MINUS) were diluted 1:5 in antibody diluent from the kit and applied to the cells for 1 hour at 37°C. Following washing with buffer A, ligation was performed using ligation buffer supplemented with ligase (1:40 dilution) and incubated for 30 min at 37°C. Cells were then washed and subjected to amplification using an amplification buffer with polymerase (1:80 dilution) for 100 min at 37°C. After two final washes for 10 min each in buffer B, the nuclei were counterstained with DAPI, as described in the immunostaining section.

### nLC-MS/MS–based proteomic analysis

For tissue samples, liver, kidney, and lung were homogenized in radioimmunoprecipitation assay buffer and centrifuged at 14,000*g* for 15 min to obtain protein lysates. Protein extracts were purified and enzymatically digested using a filter-aided sample preparation protocol. For CM samples, RAW264.7 cells were cultured in serum-free, phenol red–free MEM (Gibco) and stimulated with LPS, with or without CTM, for 24 hours. CM was concentrated using Amicon Ultra-4 centrifugal filters (Sigma-Aldrich), and proteins were precipitated with ice-cold trichloroacetic acid. Following centrifugation, the pellet was washed with cold acetone, air-dried, and digested in solution with trypsin after reduction with DTT and alkylation with iodoacetamide. For streptavidin pull-down samples, streptavidin-conjugated beads were boiled in SDS sample buffer at 95°C for 10 min. Eluates were loaded onto SDS-PAGE, and electrophoresis was stopped before protein separation. The entire protein content was excised as a single gel band for downstream processing (*75*). Proteins were then reduced, alkylated, and digested with trypsin. For SDS-PAGE gel band analysis, gel slices were excised, reduced with DTT, alkylated with acrylamide, and subjected to overnight trypsin digestion at 37°C (Worthington Biochemical Corporation, Lakewood, NJ, USA). For WCL and AGG samples, the pellets were resuspended in 4% SDS/50 mM tris-HCl (pH 8.5) and heated at 95°C for 10 min. Proteins were then reduced with DTT, alkylated with iodoacetamide, and processed using the SP3 method (*76*), followed by trypsin digestion. All samples were analyzed by nLC-MS/MS using nanoelectrospray ionization on a spray column (NTCC-360, 0.075-mm inside diameter, 150-mm length, 3-μm particle size; Nikkyo Technos Co. Ltd., Tokyo, Japan). The mobile phases consisted of 0.1% formic acid in water (solvent A) and 0.1% formic acid in 80% acetonitrile (solvent B). Mass spectrometric analysis was performed using either a Q Exactive HF-X system coupled with an Easy-nLC 1200 (Thermo Fisher Scientific) operating in data-dependent acquisition (DDA) mode (Top 20), or an Orbitrap Exploris 240 system coupled with a Vanquish Neo UHPLC (Thermo Fisher Scientific) operating in data-independent acquisition (DIA) mode. Acquisition parameters included an MS1 scan range of 500 to 740 mass/charge ratio (*m*/*z*), MS2 scan range of 200 to 1800 *m*/*z*, automatic gain control target of 3 × 10^6^, isolation window of 4 *m*/*z*, and Orbitrap resolutions of 15,000 (MS1) and 45,000 (MS2), respectively. DDA data were analyzed using Proteome Discoverer software (v3.0) with the MASCOT search engine (v2.8; Matrix Science, London, UK). DIA data were processed using Proteome Discoverer (v3.1) with the Chimerys search engine (Thermo Fisher Scientific). Database searches were performed against the *Mus musculus* SwissProt database using the following parameters: enzyme specificity, trypsin; fixed modifications, carbamidomethylation or propionamidation of cysteine residues; variable modifications, N-terminal acetylation, methionine oxidation, and pyroglutamate formation from N-terminal glutamine. Mass tolerances were set to 15 parts per million (ppm) for precursor ions and ± 30 milli mass unit (mmu) (DDA) or 20 ppm (DIA) for fragment ions. Monoisotopic precursor mass values were used, allowing up to two or three missed cleavages. Label-free quantification was performed within the Proteome Discoverer environment. Human liver samples were analyzed by SWATH-DIA MS, and data were processed using DIA-NN with a false discovery rate <1% at the protein level as previously described (*77*).

### Knowledge-based pathway analysis

Canonical pathway and upstream regulator analyses were performed using the knowledge-based functional analysis platform IPA (Ingenuity Systems, Mountain View, CA, USA) as previously described (*78*). GO enrichment analysis was conducted using the clusterProfiler R package (version 4.5.0) or Database for Annotation, Visualization, and Integrated Discovery Bioinformatics Resources 2021 (https://davidbioinformatics.nih.gov/). The GO terms were categorized into three functional domains: biological processes, molecular functions, and cellular components. GSEA was performed using the gseKEGG function in the ClusterProfile package. Genes were ranked on the basis of log_2_ fold-change values, and KEGG pathway gene sets were retrieved using the parameter organism = “mmu” for mouse. The enrichment analysis applied a weighted Kolmogorov-Smirnov–like statistic to assess the nonrandom accumulation of pathway genes at the extremes of the ranked list. Predicted TF activities were inferred using the VIPER algorithm applied to the DEGs based on the mouse DoRothEA regulon with confidence levels A to C. Results were visualized using the ggplot2 and GOplot R packages.

### IDR prediction and feature calculation

The full-length protein sequences of the mouse WCL and AGG gene lists were retrieved as mouse-specific UniProt FASTA entries using the UniProt REST API. IDR regions were predicted on the basis of AlphaFold2-derived pLDDT scores, where residues with ⟨pLDDT⟩ >0.8, ⟨pLDDT⟩ <0.7, and 0.7≤ ⟨pLDDT⟩ ≤0.8 were classified as folded, disordered, and gap regions, respectively, as previously described (*79*). Disordered segments longer than 10 amino acids were extracted for downstream analysis. IDR-specific physicochemical features, including molecular weight, hydrophobicity, charge at pH 7, isoelectric point, aromaticity, instability index, aliphatic index, Boman index, and peptide length, were calculated using the Peptides R package. Aromaticity was computed using a custom function that quantified the proportion of aromatic residues (F, W, and Y) in each IDR sequence.

### Transmission electron microscopy

Cells were fixed in 2.5% glutaraldehyde in 0.1 M phosphate buffer (PB) for 2 hours and washed with 0.1 M PB. Subsequently, cells were postfixed in 1% OsO4 buffered with 0.1 M PB for 2 hours, dehydrated in a graded series of ethanol, and embedded in Epon 812. Ultrathin sections (70 nm) were collected on copper grids, double stained with uranyl acetate and lead citrate, and examined using a transmission electron microscope (JEM-1400Flash; JEOL, Japan).

### Immuno-electron microscopy

For immunogold labeling, cells were fixed with 4% PFA and 0.1% glutaraldehyde in 0.1 M PB for 15 min at RT, followed by washing and incubation in 4% PFA for 30 min. After permeabilization with block solution (50 mM NH_4_Cl, 0.1% saponin, and 1% BSA in PB), cells were incubated with primary antibody against Rab27a (1:200, Cell Signaling Technology, 69295) and subsequently with a 1.4-nm Nanogold-conjugated goat anti-rabbit immunoglobulin G Fab′ fragment (Nanoprobes). Silver enhancement was performed using a pre-embedding immunogold protocol as previously described (*80*).

### Data mining

Gene expression data for the GSE141864 cohort were downloaded from the Gene Expression Omnibus (GEO). This dataset comprises samples from the heart, lung, kidney, liver, and spleen tissues of five patients who developed meningococcal septic shock and two control individuals who died from acute noninfectious causes.

### Statistical analysis

All quantitative data are presented as means ± SD of at least two biological replicates. Statistical analysis was performed using Student’s *t* test or Kruskal-Wallis test using GraphPad Prism 9.5.1 (GraphPad Software) or Microsoft Excel. A *P* value < 0.05 was considered significant.
