# Supplementary material for: Transglutaminase 2 regulates vimentin-dependent proteostasis during macrophage activation
Source: Sci Adv. 2026 Aug 28;12(35):eaea7513. doi: 10.1126/sciadv.aea7513 (PMC13524051; doi:10.1126/sciadv.aea7513)
Supplement: Supplementary file 1 [file sciadv.aea7513_sm.pdf]

Science Advances
AAAS

Supplementary Materials for

Transglutaminase 2 regulates vimentin-dependent proteostasis during macrophage activation

Yali Xu *et al.*

Corresponding author: Wenkui Yu, yudrnj@nju.edu.cn; Xian-Yang Qin, qin.xian-yang.p5@f.gifu-u.ac.jp

Sci. Adv. **12**, eaea7513 (2026)
DOI: 10.1126/sciadv.aea7513

The PDF file includes:

Figs. S1 to S6
Table S1
Legends for data S1 to S9

Other Supplementary Material for this manuscript includes the following:

Data S1 to S9

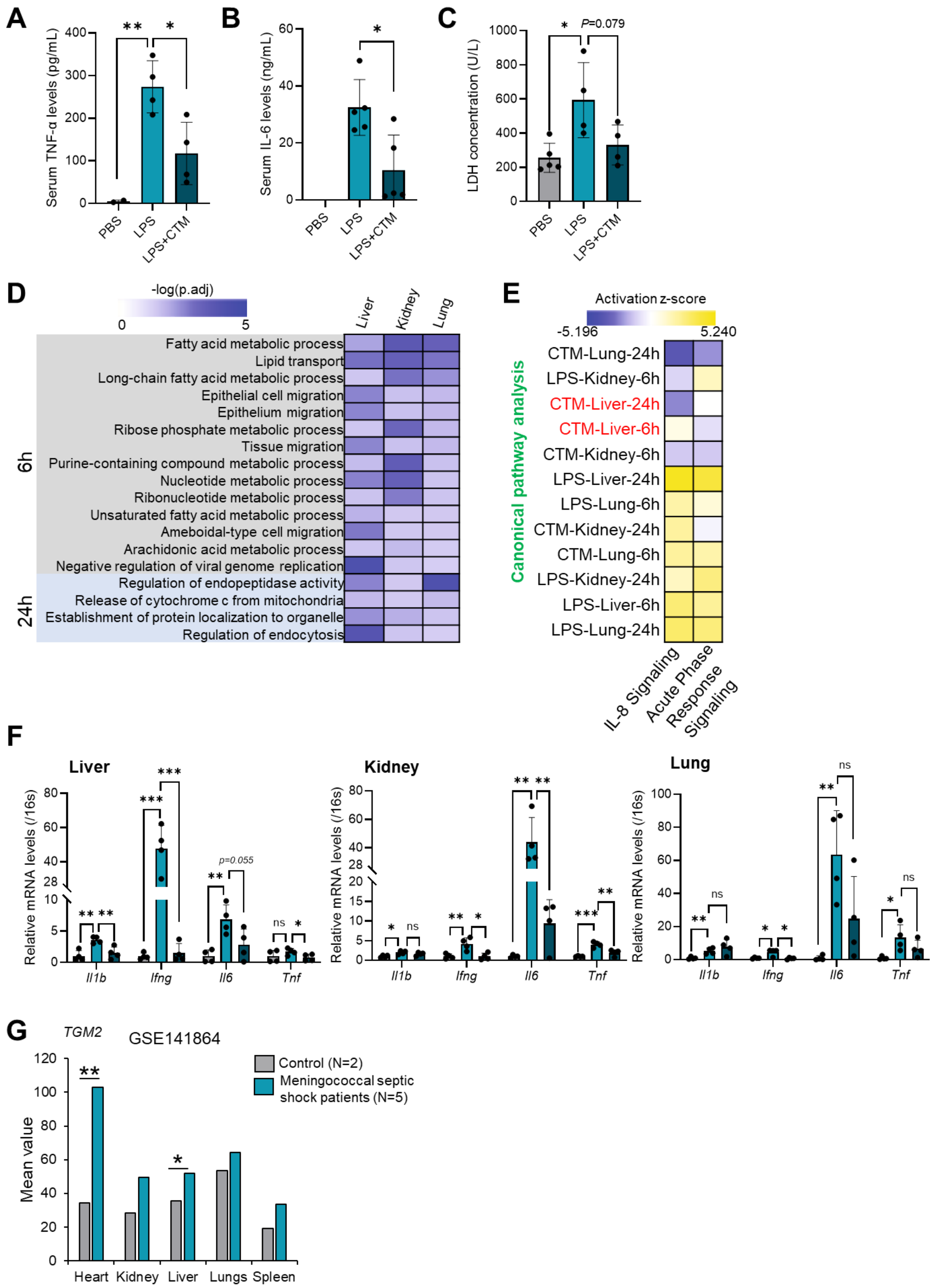

Fig. S1.

CTM improves sepsis survival by modulating hepatic inflammation and multiorgan immune responses, related to Fig 1**.** (A–C) Levels of (A) TNF-α, (B) IL-6, and (C) lactate dehydrogenase (LDH) in serums of young mice at 24 h after LPS treatment. (D) GO enrichment of biological processes shared across tissues. (E) Canonical pathway analysis (Ingenuity Pathway Analysis) of

differentially expressed proteins in liver, kidney, and lung from LPS-treated mice ± CTM. Negative z-scores indicate pathway inhibition; positive z-scores indicate activation. (F) Relative gene expression of inflammatory genes in aged mice at 6 h after LPS treatment. (G) Relative gene expression of *TGM2* in human tissues from GSE141864. $^{*}P < 0.05$, $^{**}P < 0.01$, $^{***}P < 0.001$, Student's *t*-test; ns, not significant.

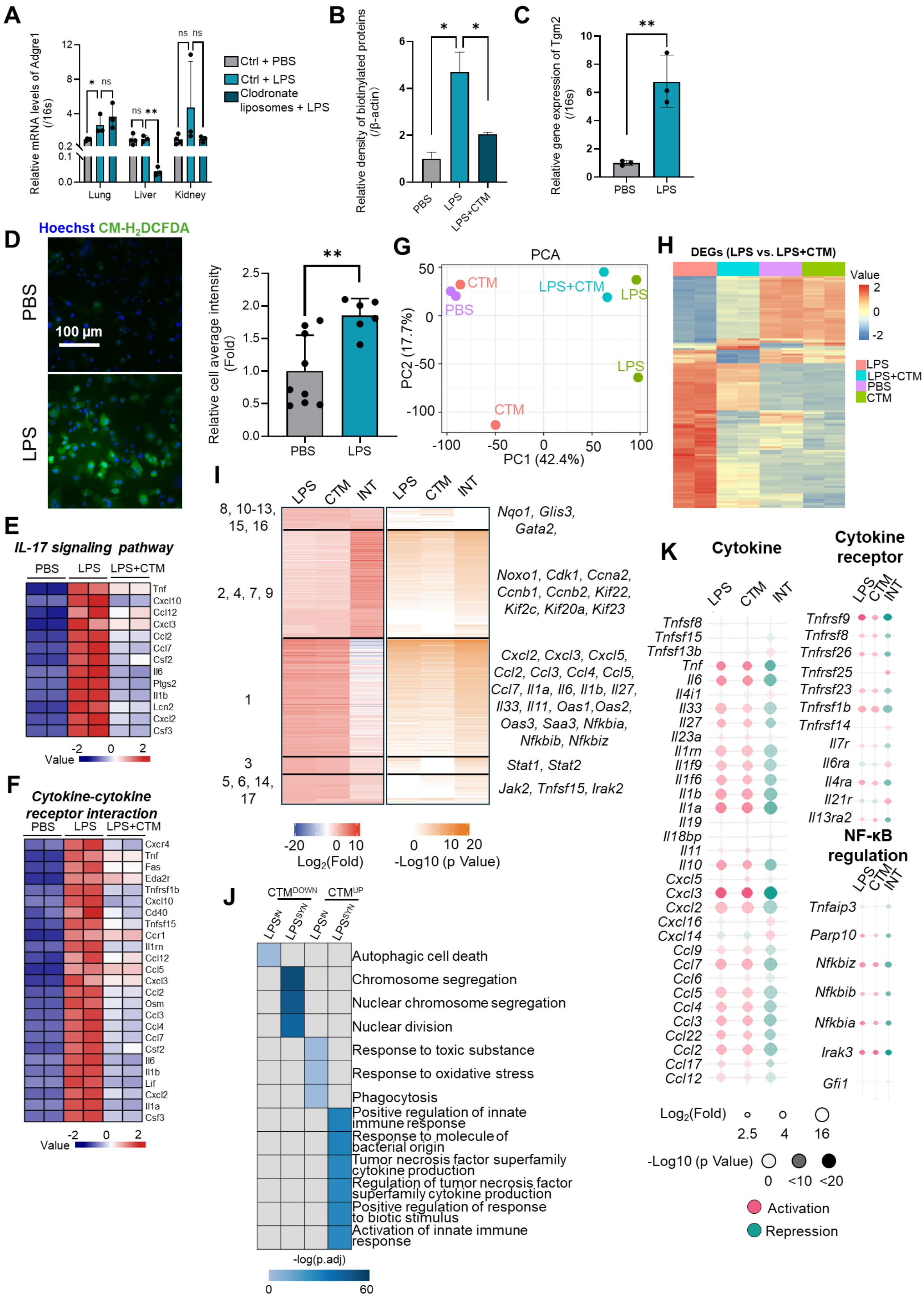

A
Relative mRNA levels of Adgre1 (/16s)
Lung
Liver
Kidney
Ctrl + PBS
Ctrl + LPS
Clodronate liposomes + LPS
B
Relative density of biotinylated proteins (/β-actin)
PBS
LPS
LPS+CTM
C
Relative gene expression of Tgm2 (/16s)
PBS
LPS
D
Hoechst CM-H2DCFDA
PBS
LPS
100 µm
Relative cell average intensity (Fold)
G
PCA
PC1 (42.4%)
PC2 (17.7%)
CTM
LPS+CTM
LPS
PBS
H
DEGs (LPS vs. LPS+CTM)
Value
LPS
LPS+CTM
PBS
CTM
E
IL-17 signaling pathway
PBS
LPS
LPS+CTM
Tnf
Cxcl10
Ccl12
Cxcl3
Ccl2
Ccl7
Csf2
Il6
Ptgs2
Il1b
Lcn2
Cxcl2
Csf3
Value
F
Cytokine-cytokine receptor interaction
PBS
LPS
LPS+CTM
Cxcr4
Tnf
Fas
Eda2r
Tnfrsf1b
Cxcl10
Cd40
Tnfsf15
Ccr1
Il1rn
Ccl12
Ccl5
Cxcl3
Ccl2
Osm
Ccl3
Ccl4
Ccl7
Csf2
Il6
Il1b
Lif
Cxcl2
Il1a
Csf3
Value
I
LPS
CTM
INT
8, 10-13, 15, 16
2, 4, 7, 9
1
3
5, 6, 14, 17
Nqo1, Glis3, Gata2,
Noxo1, Cdk1, Ccna2, Ccnb1, Ccnb2, Kif22, Kif2c, Kif20a, Kif23
Cxcl2, Cxcl3, Cxcl5, Ccl2, Ccl3, Ccl4, Ccl5, Ccl7, Il1a, Il6, Il1b, Il27, Il33, Il11, Oas1, Oas2, Oas3, Saa3, Nfkbia, Nfkbib, Nfkbiz
Stat1, Stat2
Jak2, Tnfsf15, Irak2
Log2(Fold)
-Log10 (p Value)
J
CTM DOWN
CTM UP
LPS IN
LPS SYN
Autophagic cell death
Chromosome segregation
Nuclear chromosome segregation
Nuclear division
Response to toxic substance
Response to oxidative stress
Phagocytosis
Positive regulation of innate immune response
Response to molecule of bacterial origin
Tumor necrosis factor superfamily cytokine production
Regulation of tumor necrosis factor superfamily cytokine production
Positive regulation of response to biotic stimulus
Activation of innate immune response
-log(p.adj)
K
Cytokine
LPS
CTM
INT
Tnfsf8
Tnfsf15
Tnfsf13b
Tnf
Il6
Il4i1
Il33
Il27
Il23a
Il1rn
Il1f9
Il1f6
Il1b
Il1a
Il19
Il18bp
Il11
Il10
Cxcl5
Cxcl3
Cxcl2
Cxcl16
Cxcl14
Ccl9
Ccl7
Ccl6
Ccl5
Ccl4
Ccl3
Ccl22
Ccl2
Ccl17
Ccl12
Cytokine receptor
Tnfrsf9
Tnfrsf8
Tnfrsf26
Tnfrsf25
Tnfrsf23
Tnfrsf1b
Tnfrsf14
Il7r
Il6ra
Il4ra
Il21r
Il13ra2
NF-κB regulation
Tnfaip3
Parp10
Nfkbiz
Nfkbib
Nfkbia
Irak3
Gfi1
Log2(Fold)
-Log10 (p Value)
Activation
Repression

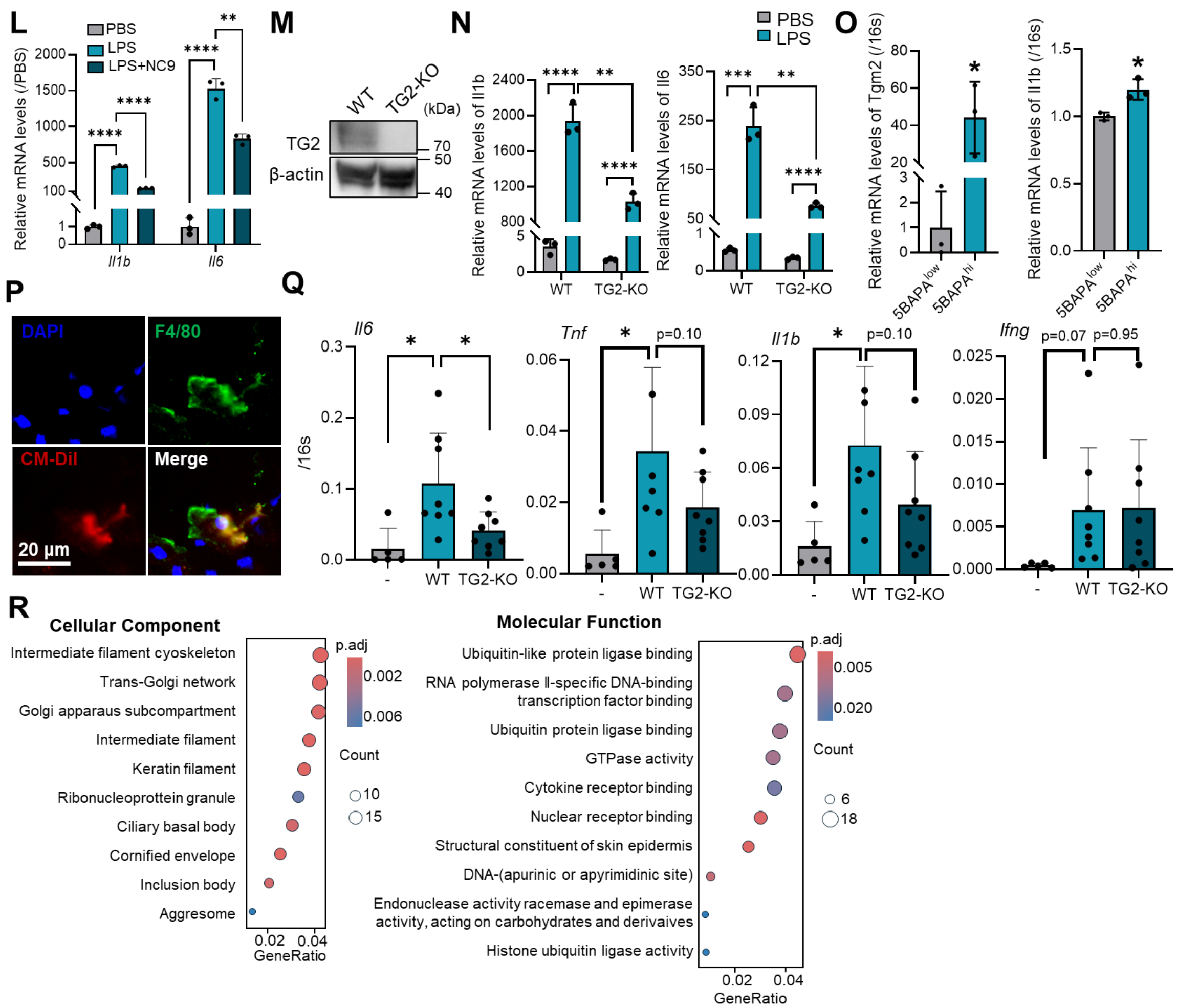

Fig. S2.

CTM suppresses LPS-induced inflammation in macrophages, related to Fig 2. (A) Macrophage marker *Adgre1* gene expression (RT-qPCR). (B) Bar graph indicated the biotinylated protein levels, normalized to β-actin. (C) Relative gene expression of *Tgm2* in LPS-treated RAW264.7 cells. (D) (Left) Double immunofluorescence staining for Hoechst 33342 (blue) and CM-H_2DCFDA (green) in RAW264.7 cells stimulated with 1 µg/ml LPS for 12 h. (Right) Quantification of fluorescence intensity of CM-H_2DCFDA. (E, F) Heatmap of core enrichment genes from GSEA terms (E) "IL-17 signaling" and (F) "Cytokine-cytokine receptor interaction". (G) PCA of RNA-seq profiles from RAW264.7 cells treated with PBS, LPS, LPS + CTM or CTM. (H) Heatmap of differentially expressed genes (DEGs; LPS vs. LPS + CTM) across all four conditions. (I) (Left) Heatmap of genes with model-inferred expression components and (Right) p value. (J) GO biological process enrichment of LPS-insensitive, or LPS-synergistic genes. (K) Expression components of gene groups. (L) Relative gene expression of *Il1b* and *Il6* in LPS-treated RAW264.7 cells ± NC9 (50 µM). (M) Immunoblots of TG2 protein levels. (N) Relative gene expression of *Il1b* and *Il6* in LPS-treated WT and TG2-KO RAW264.7 cells. (O) Relative gene expression of *Tgm2* and *Il1b* in sorted $5BAPA^{hi}$ and $5BAPA^{low}$ RAW264.7 cells. (P) Triple immunofluorescence staining for DAPI (blue), F4/80 (green), and CM-Dil (red) in the liver. (Q) Relative gene expression of *Il6*, *Tnf*, *Il1b*, and *Ifng* in the livers of septic mice reconstructed with WT or TG2-KO RAW264.7 cells.

(R) Top 10 enriched GO cellular component and molecular function terms for CTM-regulated proteins. *P < 0.05, **P < 0.01, ***P < 0.001, ****P < 0.0001, Student's *t*-test; ns, not significant.

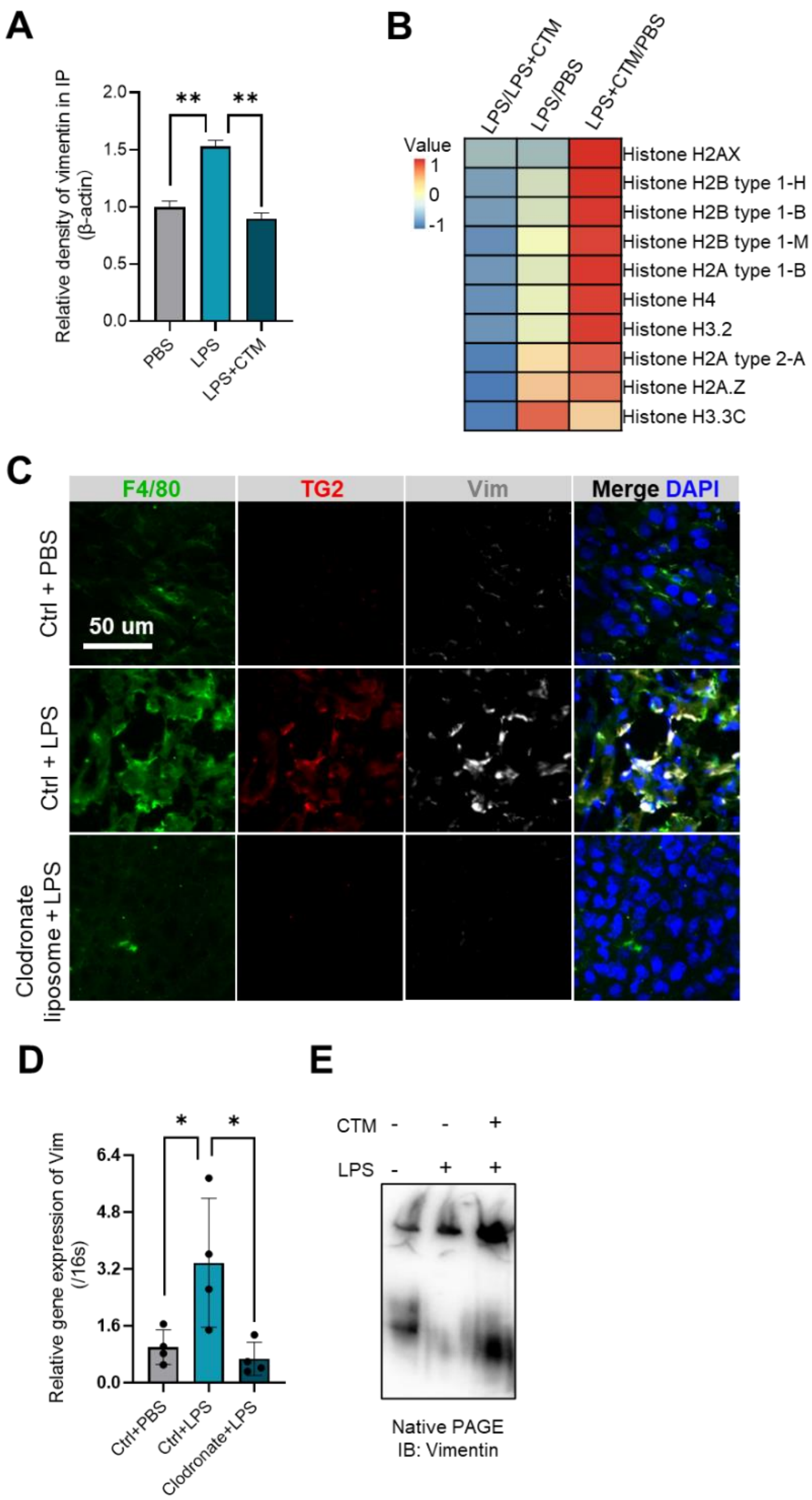

Fig. S3.

Macrophage depletion reduces vimentin expression in septic mice, related to Fig 3. (A) Bar graph indicated the vimentin levels in immunoprecipitates, normalized to β-actin. (B) Heatmap of normalized abundance of core histones identified by nLC-MS/MS from streptavidin pull-down eluates. (C) Quadruple staining of DAPI (blue), F4/80 (green), TG2 (red), and Vim (gray) in liver after clodronate liposome treatment. (D) Relative gene expression of *Vim* in liver. (E) Analysis of vimentin abundance in RAW264.7 cells by NativePAGE and western blot. *$P < 0.05$, **$P < 0.01$, Student's *t*-test.

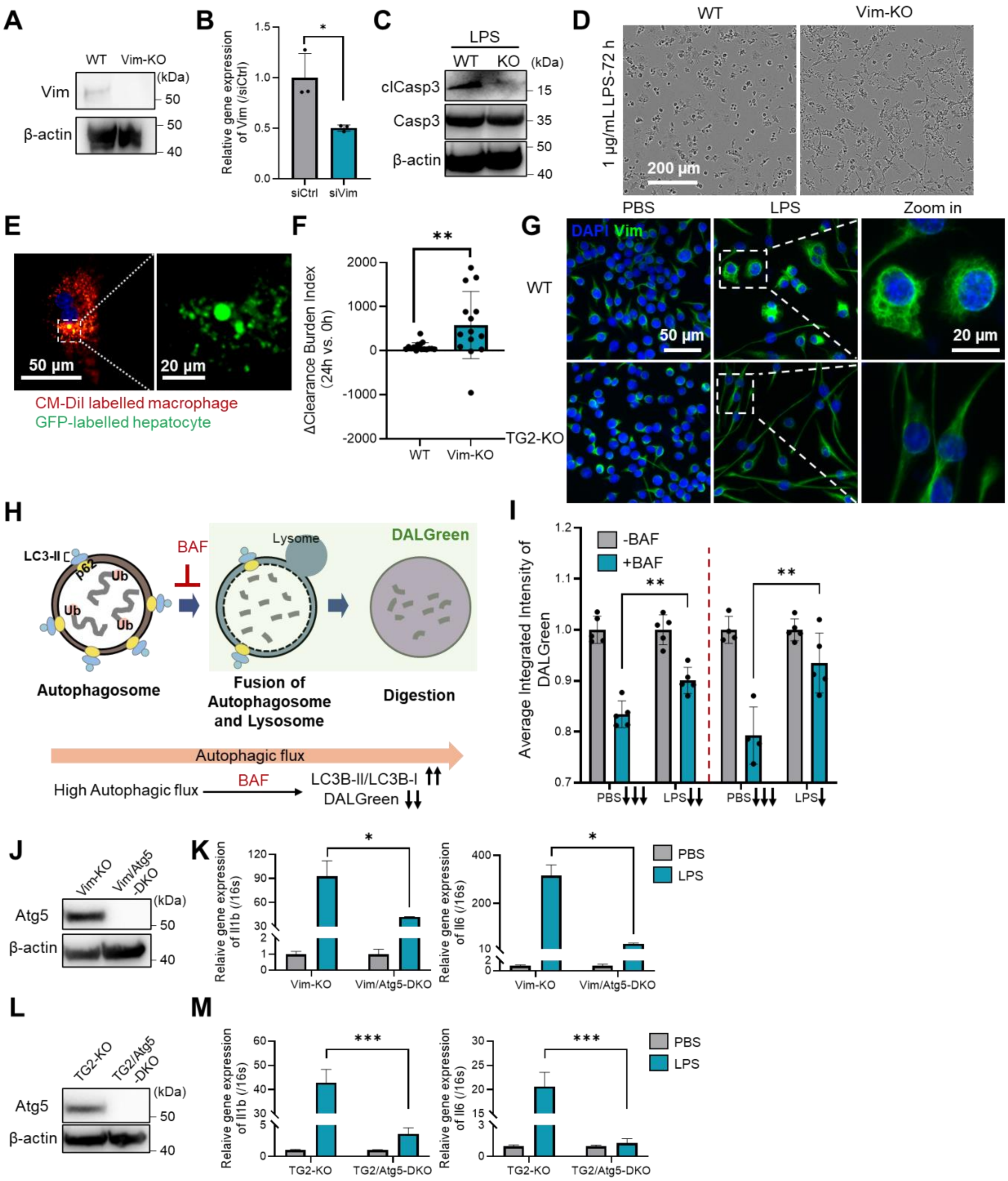

Fig. S4.

Vim deficiency attenuates inflammation and enhances proteasomal proteostasis, related to Fig 4. (A) Immunoblots of Vim expression. (B) Relative gene expression of *Vim* in RAW264.7 cells transfected with 200 nM siCtrl or siVim for 24 h. (C) Immunoblots of caspase-3 (Casp3) and cleaved caspase-3 (clCasp3) in WT and Vim-KO RAW264.7 cells treated with 1 μg/ml LPS for 72 h. (D) Representative phase-contrast images of LPS-treated WT and Vim-KO RAW264.7 cells treated with 1 μg/ml LPS for 72 h. (E) Representative microscopic images of co-culture of CM-

Dil-stained RAW264.7 cells (red) and GFP-labelled hepatocytes. (F) Quantification of Δ clearance burden index. (G) Double immunofluorescence staining of DAPI (blue) and Vim (green) in LPS-treated WT and TG2-KO RAW264.7 cells. (H) Schematic illustration depicting the effects of bafilomycin A1 (BAF) treatment on autophagic flux. (I) Autophagic flux measured by DALGreen fluorescence intensity. (J, L) Immunoblots of Atg5 expression. (K, M) Relative gene expression of *Il1b* and *Il6* in LPS-treated Vim-KO and TG2-KO RAW264.7 cells. $^{*}P < 0.05$, $^{**}P < 0.01$, $^{***}P < 0.001$, Student's *t*-test.

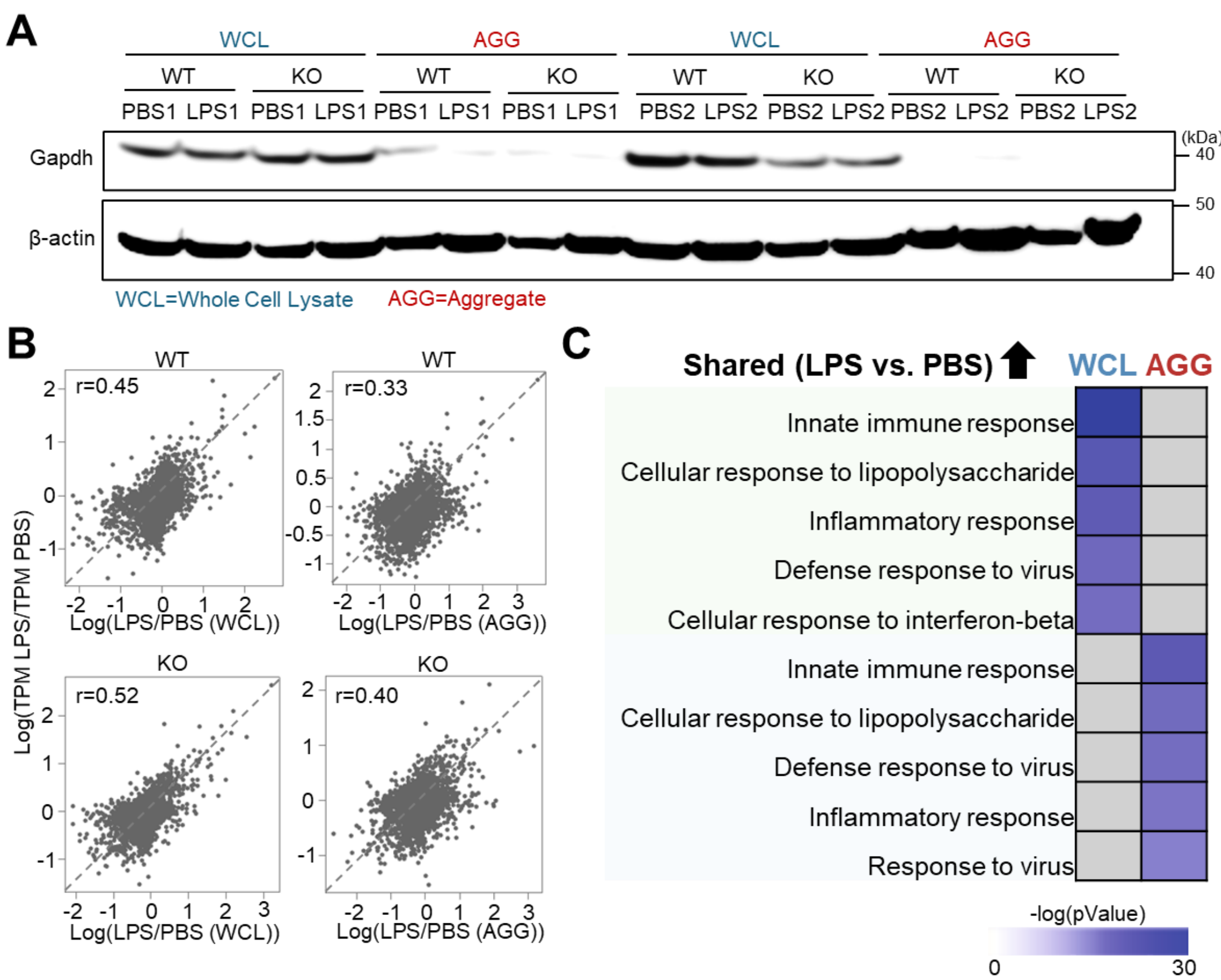

Fig. S5.

Vim-dependent alterations in protein abundance and aggregation, related to Fig 5. (A) Immunoblots of Gapdh and β-actin in AGG and WCL fractions from WT and Vim-KO cells. Samples correspond to those analyzed by silver staining in Fig 5B. (B) Correlation plots of log10 (fold change) in protein abundance in WCL and AGG fractions (nLC-MS/MS) vs. transcript levels (RNA-seq). r, Pearson's correlation coefficient. (C) Top 5 enriched GO biological processes for LPS-upregulated proteins in WCL and AGG fractions shared between WT and KO cells.

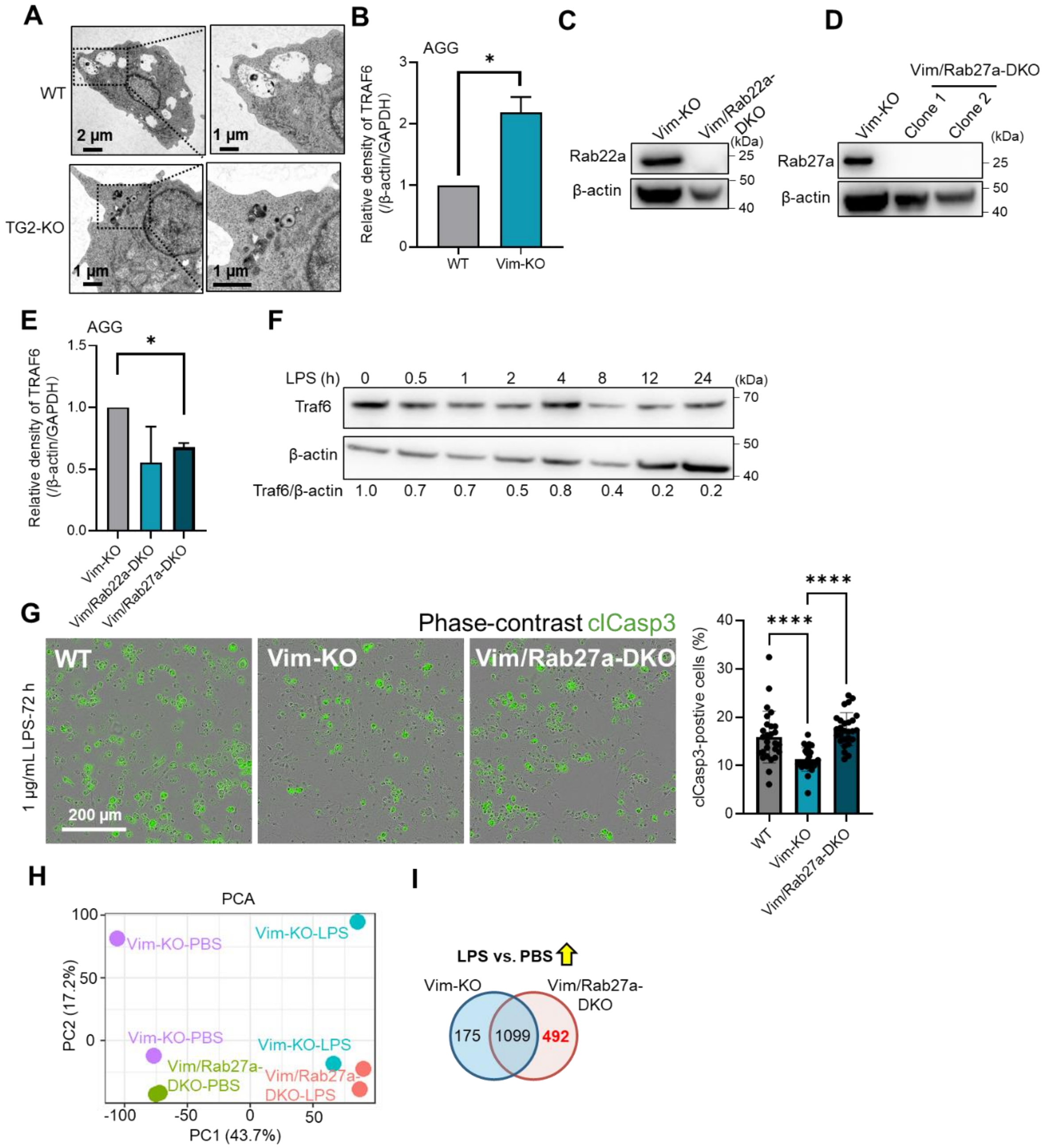

Fig. S6.

Vim deficiency promotes proteasomal degradation of Traf6 aggregates, related to Fig 6. (A) TEM images of LPS-treated WT and TG2-KO RAW264.7 cells. Right: magnified views. Scale bars: 1 µm. (B) Bar graph indicated Traf6 levels in AGG fractions from LPS-treated WT and Vim-KO RAW264.7 cells, normalized to β-actin and GAPDH. (C, D) Immunoblots of (C) Rab22a and (D) Rab27a expression. (E) Bar graph indicated Traf6 levels in AGG fractions from LPS-treated Vim-KO, Vim/Rab22a-DKO, and Vim/Rab27a-DKO RAW264.7 cells, normalized to β-actin and GAPDH. (F) Immunoblots of Traf6 in LPS-treated WT RAW264.7 cells. (G) (Left) Representative overlay images of phase-contrast and cleaved caspase-3 (clCasp3; green) fluorescence. (Right) Quantification of the percentage of clCasp3-positive cells in WT, Vim-KO, and Vim/Rab27a-

DKO cells treated with 1 μg/ml LPS for 72 h. (H) PCA of RNA-seq profiles from Vim-KO and Vim/Rab27a-DKO cells treated with PBS or 1 μg/ml LPS for 12 h . (I) Venn diagram of genes significantly upregulated by LPS stimulation (fold change > 2 and adjusted p-value < 0.05). *P < 0.05, ****P < 0.0001, Student's *t*-test.

Table S1.

Primer sequences for real-time PCR.

Species	Gene	Forward (5' towards 3')	Reverse (5' towards 3')
Mouse	Adgre1	TGTCTGACAATTGGGATCTGCCCT	ATAGCTTCCGAGAGTGTTGTGGCA
Mouse	16s	AGGAGCGATTTGCTGGTGTGGA	GCTACCAGGGCCTTTGAGATGGA
Mouse	Il6	TTCCATCCAGTTGCCTTCTTGG	TTCTCATTTCCACGATTTCCCAG
Mouse	Ifng	ACAGCAAGGCGAAAAAGGATG	TGGTGGACCACTCGGATGA
Mouse	Il1b	GCCCATCCTCTGTGACTCAT	AGGCCACAGGTATTTTGTCG
Mouse	Tnf	TGTCTACTCCCAGGTTCTCT	GGGGCAGGGGCTCTTGAC
Mouse	Ccl2	GCCCCACTCACCTGCTGCTACT	CCTGCTGCTGGTGATCCTCTTGT
Mouse	Tgm2	CTAGAGGCTTCTACTGGCTACC	GCGTAAGGACATATTCCCGTC
Mouse	Lcn2	CACACTCACCACCCATTCAG	TGGAAGAACCAAGGAGCTGT
Mouse	Vim	CGTCCACACGCACCTACAG	GGGGGATGAGGAATAGAGGCT
Human	ACTB	GCACAGAGCCTCGCCTT	GTTGTCGACGACGAGCG
Human	IL6	AAAGAGGCACTGGCAGAAAA	ATGAGGAGACTTGCCTGGTG
Human	TNF	TCCTTCAGACACCCTCAACC	AGGCCCCAGTTTGAATTCTT
Human	IL1B	CAGCCAATCTTCATTGCTCA	GCATCTTCCTCAGCTTGTCC

Data S1.
Raw dataset from nLC-MS/MS-based proteomic analysis of mouse liver, lung, and kidney tissues.

Data S2.
Raw dataset from RNA-seq analysis of mouse liver tissues.

Data S3.
Raw dataset from RNA-seq analysis of RAW264.7 cells.

Data S4.
Raw dataset from nLC-MS/MS-based proteomic analysis of conditioned medium.

Data S5.
Raw dataset from nLC-MS/MS-based proteomic analysis of lysates pulled down using streptavidin-conjugated beads.

Data S6.
Raw dataset from nLC-MS/MS-based proteomic analysis of protein bands excised from SDS-PAGE.

Data S7.
Raw dataset from RNA-seq analysis of WT and Vim-KO RAW264.7 cells.

Data S8.
Raw dataset from nLC-MS/MS-based proteomic analysis of WCL and AGG fractions in WT and Vim-KO RAW264.7 cells.

Data S9.
Raw dataset from RNA-seq analysis of Vim-KO and Vim/Rab27a-DKO RAW264.7 cells.
